# Micro plastic driving changes in the soil microbes and lettuce growth under the influence of heavy metals contaminated soil

**DOI:** 10.3389/fpls.2024.1427166

**Published:** 2024-09-11

**Authors:** Jazbia Shirin, Yongjing Chen, Azhar Hussain Shah, Yanmei Da, Guowei Zhou, Qingye Sun

**Affiliations:** ^1^ Anhui Province Engineering Laboratory for Mine Ecological Remediation, School of Resources and Environmental Engineering, Anhui University, Hefei, Anhui, China; ^2^ Anhui Province Key Laboratory of Wetland Ecological Protection and Restoration, School of Resources and Environmental Engineering, Anhui University, Hefei, Anhui, China; ^3^ Department of Biotechnology and Genetic Engineering, Hazara University, Mansehra, Pakistan

**Keywords:** polystyrene microplastic, heavy metals, microbes, *Lactuca sativa*, 16S rRNA sequencing

## Abstract

Microplastics (MPs) have garnered global attention as emerging contaminants due to their adaptability, durability, and robustness in various ecosystems. Still, studies concerning their combination with heavy metals (HMs), their interactions with soil biota, and how they affect soil physiochemical properties and terrestrial plant systems are limited. Our study was set to investigate the combined effect of HMs (cadmium, arsenic, copper, zinc and lead) contaminated soil of Tongling and different sizes (T1 = 106 µm, T2 = 50 µm, and T3 = 13 µm) of polystyrene microplastics on the soil physiochemical attributes, both bacterial and fungal diversity, compositions, AMF (arbuscular mycorrhizal fungi), plant pathogens in the soil, and their effect on *Lactuca sativa* by conducting a greenhouse experiment. According to our results, the combination of HMs and polystyrene microplastic (PS-MPs), especially the smaller PS-MPs (T3), was more lethal for the lettuce growth, microbes and soil. The toxicity of combined contaminants directly reduced the physio-biochemical attributes of lettuce, altered the lettuce’s antioxidant activity and soil health. T3 at the final point led to a significant increase in bacterial and fungal diversity. In contrast, overall bacterial diversity was higher in the rhizosphere, and fungal diversity was higher in the bulk soil. Moreover, the decrease in MPs size played an important role in decreasing AMF and increasing both bacterial and fungal pathogens, especially in the rhizosphere soil. Functional prediction was found to be significantly different in the control treatment, with larger MPs compared to smaller PS-MPs. Environmental factors also played an important role in the alteration of the microbial community. This study also demonstrated that the varied distribution of microbial populations could be an ecological indicator for tracking the environmental health of soil. Overall, our work showed that the combination of HMs and smaller sizes of MPs was more lethal for the soil biota and lettuce and also raised many questions for further studying the ecological risk of PS-MPs and HMs.

## 1 Introduction

The post-industrialization era saw a sharp rise in the manufacture of plastics worldwide, which was required at that time. Global plastic production reached 368 million tons in 2019 and is predicted to triple within 20 years, and the COVID-19 epidemic drove up plastic product production even more, reaching 390.7 million tons in 2021. Asia is the world’s largest plastic producer, with 187.68 million of the 368 million tons produced worldwide, followed by Europe with 58.88 million tons. However, only 26% of all plastic manufactured is recycled, with the remaining waste either ending up in landfills or contaminating the environment in other ways (Azeem et al., 2022; Huang et al., 2021; Plastics Europe, 2019; Lebreton and Andrady (2019); Plastics Europe, 2022; An et al., 2023. Furthermore, it is anticipated that by 2050, the amount of plastic trash generated will increase to 12,000 million tons (Geyer et al., 2017; Ayeleru et al., 2020; Huang et al., 2023).

Microplastics, small polymer particles less than 5 mm, are a growing environmental contaminant with widespread detection and potential hazards, prompting research in ecological science and emerging pollutants (de Souza MaChado et al., 2018; Zhao et al., 2023; Peng et al., 2018; Galloway et al., 2017). Research on microplastics’ effects on soil and plant development is limited and the majority of the research is currently conducted on aquatic environments (Peng et al., 2018; Qi et al., 2018; Zhao et al., 2020). Soils may represent an extensive reservoir of microplastics (Bläsing, and Amelung, 2018; Hurley and Nizzetto, 2018), and the amount of MPs pollution on land may be 4–23 times greater than that in the ocean (Horton et al., 2017). MPs significantly alter soil’s physical and chemical properties, decreasing organic matter and microbial activity and posing significant threats to plant communities and ecosystem stability (Khalid et al., 2022; Rillig, 2012). Polystyrene (PS) is one of the most widely used types of plastic and is hydrophobic, and can easily be absorbed on the root surface, thus decreasing root growth (Loos et al., 2014; Nel et al., 2009; Pang et al., 2023; Xiong et al., 2023).

Biodegradable microplastic produces a stronger impact on arbuscular mycorrhizal fungal (AMF) diversity and community (Wang et al., 2017; Bouaicha et al., 2022; Chen et al., 2022d). According to recent research, the primary influence of MP on plants is felt in the roots, which are then followed by the leaves, shoots, and stems (de Souza MaChado et al., 2019; Zhang et al., 2020a). Moreover, microplastic-driven changes in soil properties, plant growth, and development are highly dependent on MPs type, dose and exposure time (Ossowicki, 2020; de Souza MaChado et al., 2018; Wang C. et al., 2019; Medyńska-Juraszek and Jadhav, 2022; Jiang et al., 2019; Wang et al., 2020; Ge et al., 2021) and small particle sizes of MPs can penetrate deeper soil layers, causing more harm to rhizosphere soil microbes and affecting soil nutrients and plant growth (Dong et al., 2021). Evidence also shows that low doses of MPs have negligible effects on soil organisms (Rodriguez-Seijo et al., 2017; Rodríguez-Seijo and Pereira, 2019). Still, research on the interactions between MPs, NPs, and higher plants is scarce (Rillig, 2012).

MPs and NPs with coexisting contaminants affect soil properties, structure, function, growth performance, and biology and potentially trigger physiology and genotoxicity (Yu Z., et al., 2021; de Souza MaChado et al., 2018; Rillig, 2012; Xiang et al., 2022). The complicated MPs and HMs contamination of soils has garnered more interest (Wang et al., 2020; Dong et al., 2021, 2022; Meng et al., 2023; Zhao et al., 2023). Heavy metals are found to be attracted to MPs, and they become adsorbed to their surfaces (Khalid et al., 2021). Heavy metals like copper, lead, mercury, and zinc, found in nature or anthropogenically (such as industrialization, agriculture, mining, etc.), pose a major environmental issue, affecting soil pollution and causing toxicity to plants, requiring attention in ecological science and botany (Khalid et al., 2019; Sharma et al., 2023; Nagajyoti et al., 2010; Demková et al., 2019; Chen et al., 2019a, b; Liu et al., 2019b; Zhang M. et al., 2019; Cui et al., 2020; Kumar et al., 2020). MPs have a large specific surface area that is rich in various functional groups, which allows them to directly adsorb metal ions and change the chemical speciation of those ions in soils (Cao et al., 2021; Kumar et al., 2022). The interaction of MPs and HMs is critical in defining the biological impacts of HMs within the soil environment. The interaction of MPs and HMs is complex and is impacted by a range of factors, including the character of MPs, the kind of HMs, and experimental circumstances (Kumar et al., 2022; An et al., 2023). Among these, experimental conditions, MPs type, and dose may also affect the combined pollution environments (An et al., 2023; Huang et al., 2023). MPs’ type, quantity, and size significantly influence HM accumulation in plants, and PE and PS promote more HM transfer and accumulation. MPs, due to their large surface area and strong hydrophobicity, play a crucial role in the accumulation and transfer of HMs in soil-plant systems and can increase the availability of HMs in soil due to their vector effects (Li et al., 2021; Wang et al., 2021; Kumar et al., 2022; Proto et al., 2023; Chen et al., 2024). Additionally, the addition of MPs altered the microbial community and plant root metabolites in rhizosphere soils, thereby facilitating the uptake of HMs by plant roots (Xu et al., 2023). Biodegradable MPs showed an inhibiting effect on Cd accumulation in plants, and MPs addition significantly increased Cd and Pb bioavailability in soil, but significantly reduced As bioavailability. MPs also showed significant promoting effects on the accumulation of Cd, Pb, and Cu in plant roots and shoots. MPs may increase the risks of these metalloids in agroecosystems by increasing their uptake in plants. Indeed, MPs addition can improve the desorption capability of Cd in soil particles, increasing the bioavailability of Cd in the soil-lettuce system. The number of observations on biodegradable MPs is limited, which may induce large variability. Therefore, the impacts of biodegradable MPs require further investigation**. (** Chen et al., 2024; Wang et al., 2022 **).**

The Tongling area in China is renowned for its abundant Cu resources, dating back over 3000 years during the Shang and Zhou dynasties (Zhang et al., 2020a), with the first Cu mine at Tongguanshan operating since 1949 (Shen et al., 2017). (Wang J. et al., 2019) also revealed that the greatest impact on human health in this region by Cr, Pb, Cd, and Cu cannot be ignored. The Tongguanshan area, located 6 km from the Yangtze River, is at risk of heavy metal pollution if pollution is not controlled. The combined effect of heavy metals and MPs-NPs on terrestrial plants is urgently needed due to their interaction (Feng X. et al., 2022; Liu Y. et al., 2021). Moreover, mounting data suggests that MPs/NPs may transport potential pathogens and antibiotic resistance genes (ARGs) in soil (Ding et al., 2021; Zhu et al., 2021). Lettuce (*Lactuca sativa*) is a highly significant salad vegetable globally, owing to its high nutritional content, production potential, and financial gain (Acharya et al., 2013). It is said to have originated in the Mediterranean region and is a member of the Asteraceae family. Vitamins A and C and minerals like calcium and expectorant are abundant in lettuce (Kalloo and Parthasarthy, 2003). Due to its widespread consumption, lettuce is highly susceptible to harmful substances in the soil, making it a useful model plant for studies on phytoaccumulation (da Silva et al., 2015; Rashid et al., 2014). Because of this, lettuce is often used as a test plant for studies on pollutants (Kukier et al., 2010; Melo et al., 2012; Mehmood et al., 2013; Fontes et al., 2014; da Silva et al., 2015). The high commercial value of Lettuce makes it an important crop to be protected from the contaminants.

The current knowledge on the impacts of microplastics with the combination of HMs on soils (physiochemical characteristics and structure) and their associated biota currently remains inadequate to address the risks to the terrestrial environment fully, and to our knowledge, only a few studies have examined the effects on vascular or so-called higher plants.

To this end, we used the soil contaminated with heavy metals (HMs) and polystyrene microplastics (PS-MPs) to assess their impact on the soil microbiota and lettuce (*Lactuca sativa*), a terrestrial plant species. The objectives of this study are to (1) Analyze alterations in soil health indicators resulting from the combined presence of microplastics and heavy metals. (2) Examine the impact of microplastics and heavy metals on lettuce growth parameters/performance. (3) Assess the impact of varied microplastic sizes on soil microbial diversity and compositions in heavy metal-enriched environments. (4) Explore interactions/establish relationships between shifts in soil bacterial and fungal communities due to microplastic-heavy metal interactions and subsequent effects on the growth and health of lettuce plants. Significance: (I) The results of this study will help to improve our understanding of the soil microecology of MPs and HMs. (II) Our results provide insights into the eco-toxicological effects of microplastics on higher plants and a scientific basis for ecological risk assessments.

## 2 Methodology/materials and methods

### 2.1 Description of the study area

Tongling is located in the central part of Anhui Province. The main river in Tongling City is the Tongling section of the Yangtze River, with a total length of 55 km, 6–7 km away from the test plots. Tongling lies in the subtropical humid climate zone, with obvious monsoon characteristics. The climate is mild; the average annual temperature is 16.2°C, there is abundant rainfall, the average annual precipitation is 1390 mm, the spring and summer are rainy with sufficient sunshine, and the frost-free period is 238 days. Hence, the summer is hot, and the winter is mild. The monthly average relative humidity is 75%–81% throughout the year. Mining activity is prevalent in Tongling. Rich mineral resources, complete mineral types, and large reserves characterize the region. It is one of the famous non-ferrous metal bases and one of China’s six Cu production bases. Over the long mining period, the study area has been affected by massive pollution from the mining industry.

### 2.2 Soil Sample collection

Soil samples were collected from the copper mine tailings disposal site (N30°53′55.3″E117°55′7.0″) at depths 0 to 20 cm, which is located approximately 3 km northeast of Tongling City, Anhui Province, Southeast China. After air-drying, the soil samples were grounded and divided into two parts. One was passed through a 100-mesh nylon screen and then stored in Ziploc bags at 4°C and -80°C for further analysis, and the other part was sieved (<2 mm) and shifted to the greenhouse for the pot experiment.

### 2.3 Plant and treatments

We chose Lettuce (*Lactuca sativa*) seeds for the pot experiment and PS-MPs (polystyrene microplastic). Lettuce (*Lactuca sativa*) seeds were purchased from Hezhiyuan Seed Inc. (Shandong, China). This lettuce variety was selected due to its widespread cultivation and intrinsic characteristic of high Cd uptake (Tang et al., 2016). The polystyrene microplastic was purchased from the Zoomlion Plastics Technology Co. (Guangdong, China) and the MPs were immersed in an anhydrous ethanol (≥99.7%, Cologne, China) for disinfection and dried in an oven (BGZ-140, Boxun, China) at 50°C for later use (Xiang et al., 2023). Morphology and detailed information of PS-MPs are provided in **Supplementary Figure S1** in the supporting information. We have used MPs with three different sizes (106 µm, 50 µm, and 13 µm) as a treatment, which were denoted as T1 = 106 µm, T2 = 50 µm, T3 = 13 µm, Day 0 = soil at the time of collection, while Control (without microplastic, before sowing seeds) was denoted as C.

### 2.4 Experimental setup

Plastic pots with a height of 25 cm and diameter of 35 cm were used for this experiment, with each treatment having five replications.7kg soil was added into each pot (total 20 pots) and for each pot, 1.4 g MPs/kg soil (except for the controls) were added to pots and manually mixed, (Qi et al., 2022) watered, and kept for weeks to attain equilibrium (Liu et al., 2020a). After that, 5 seeds of uniform size were sterilized for 10 min with 2% NaClO solution, rinsed with deionized water, and sown in each pot in the greenhouse, and thinning was done after 10 days of germination to maintain three plants per pot. All other agronomic practices like irrigation, weeding, and plant protection measures were kept the same for all treatments.

### 2.5 Soil sampling

Soil samples (rhizosphere and bulk) were collected carefully after the seeds had grown for 80 days to determine the microbial community. After 110 days, plants were harvested, and soil samples were collected for soil physiochemical parameters, determination of heavy metals, and microbial community. Plant physical parameters were also calculated.

### 2.6 Soil physiochemical analyses

The pH was measured using a pH meter (Metro-pH320; Mettler Toledo Instruments Ltd., Shanghai, China) at a soil-to-water ratio of 1:2.5. Soil organic matter was measured using the K2CrO7–H2SO4 oxidation method (Ren et al., 2015). The electric conductivity (EC) of the soil samples was determined using a conductivity meter at a 1:5 soil-to-water ratio.

Soil total nitrogen (TN) was determined using Kjeldahl’s method (HJ 717—2014) (Ros et al., 2010). Potassium chloride solution extraction spectrophotometry and ultraviolet spectrophotometry were used to determine ammonium nitrogen, nitrate nitrogen, and nitrite, respectively (Ministry of Environmental Protection, PRC, 2012; Standardization Administration of China., 2016). The model of the spectrophotometer was a T6 UV-visible spectrophotometer produced by Beijing Puxi General Instrument Co., Ltd. The total phosphorus was determined by the Lu (2020) method, while the total carbon (TC) was determined by the dry combustion method (Nelson and Sommers, 1996). Total soil Cu, Pb, and Zn were determined by flame atomic absorption spectrophotometry HJ 491-2019 (Liu H. et al., 2021), and for the determination of total Cd in 0.20 g of soil were digested with a mix of 5 mL of HNO3 + 1 mL of HClO4 + 1 mL of HF and processed according to “Soil-Quality-Determination of Lead, Cadmium-Graphite Furnaces atomic absorption spectrophotometry GB/T 17141-1997 (Yang et al., 2019). The concentrations of Cd were determined using inductively coupled plasma-mass spectrometry (ICP-MS, Agilent, 7700x) following a standard procedure and total As by GB/T 22105.2-2008 (General Administration of Quality Supervision, Inspection, and Quarantine of the People’s Republic of China, 2008).

### 2.7 Plant physiochemical analyses

Different plant agronomic indicators were recorded, including plant height, diameter and length of leaves, stem, and root, fresh and dry weight of stem, leaves, and root, number of leaves, and whole plant weight.

#### 2.7.1 Relative water content and electrolyte leakage

The relative water content (RWC) of lettuce leaves was measured according to the method of Mayak et al. (2004). After weighing, fresh leaves were dipped overnight in water, and then the leaves were weighed again to get a fully turgid weight. Afterward, these were placed in an oven at 70 ± 2°C to measure the oven-dry biomass.


$$\text{Relative water content}(\text{RWC})=\frac{\text{Fresh biomass}-\text{Dry biomass}}{\text{Fully turgid biomass}-\text{Dry biomass}}\times 100$$

Electrolyte leakage (ELL) was determined according to the method described by Ahmad et al. (2014). The fully expanded leaf was cut into small pieces about 5 mm in length and placed in test tubes containing 10 mL of deionized water and then placed in a shaking incubator for four hours at 30°C, and then the electrical conductivity of the initial medium (EC1) was measured. After that, all the test tubes were placed in an autoclave at 121°C for 20 min, allowed to cool up to 25°C, and electrical conductivity (EC2) was measured and calculated by the following formula:


$$\text{Electrolyte leakage}\;(\text{ELL})=\frac{\text{EC}1}{\text{EC}2}\times 100$$

#### 2.7.2 Biochemical parameters

Total superoxide dismutase (SOD) and peroxidase (POD) in leaves were estimated using spectrophotometry at absorbance 560 nm and 470 nm, respectively. Leaves were ground with mortar while the mortar was put on the ice, and the pestle was placed in liquid nitrogen. This pattern was standardized in 0.05 M phosphate buffer (maintaining pH at 7.8), filtered through four layers of muslin cloth and centrifuged at 12,000g at 4°C for 10 min. Peroxidase (POD) activity was determined by using KIT Biosharp (product no. BL1064B), Hefei, China, and their protocols, while superoxide dismutase (SOD) activity was determined by using Kit Biosharp (product no. BL901A), Hefei, China, and following their protocols.

### 2.8 DNA extraction, PCR amplification, sequencing

DNA was extracted by using the TIANamp Soil DNA Kit (Tiangen, China), following the instructions. Microbial community compositions were characterized with multiplexed MiSeq sequencing at Shanghai Biozeron Biological Technology Co., Ltd. (Shanghai, China).

The PCRs of the bacterial 16S rRNA gene V3–V4 region were performed with the primer sets 515F (5’- GTGCCAGCMGCCGCGG-3’) and 907R (5’- CCGTCAATTCMTTTRAGTTT-3’), while PCRs of the fungal ITS region was performed with the primer sets ITS1F (5’- CTTGGTCATTTAGAGGAAGTAA-3’) and (5’- GCTGCGTTCTTCATCGATGC-3’).

#### 2.8.1 PCR amplification procedure

PCR TransStart Fastpfu DNA Polymerase (20μl): 5×FastPfu Buffer 4 μl, 2.5 mM dNTPs: 2 μl, Forward Primer (5 μM): 0.8μl, Reverse Primer (5 μM):0.8μl, FastPfu Polymerase: 0.4 μl, Template DNA: 10ng and add dd H2O to 20 μL.

#### 2.8.2 PCR reaction system

a. 1× (5 minutes at 95°C); b. cycles × (30 seconds at 95°C, 30 seconds at 55°C, 45 seconds at 72°C) c. 10 minutes at 72°C, and amplification products were detected by 1.5% agarose gel electrophoresis; finally, the pure PCR amplified samples were sequenced on the Illumina MiSeq platform at Shanghai Biozeron Technology Co., Ltd. (Shanghai, China).

### 2.9 Statistical analysis

Physicochemical variables were displayed as the mean value ± standard error (SE) in Excel 2016 (Microsoft Office 2016, Microsoft, USA). Statistical significance analysis was performed by using Duncan’s multiple range test (p < 0.05), and statistical analysis was performed via analysis of variance (ANOVA) in SPSS 22.0. The result was represented by GraphPad Prism 6.0 (GraphPad Software, Inc., San Diego, CA) (Hu et al., 2022). Bioinformatics analysis was performed on the raw sequencing data to analyze microbial community composition, diversity and function. The relationship between the microbial community and environmental factors was determined by redundancy analysis (RDA) using RStudio (version 1.2.1335) with the vegan package. Chao1 and Shannon’s indices were applied to evaluate the alpha diversity, and an online platform for data analysis was used to perform the alpha diversity analysis, the genescloud tools (https://www.genescloud.cn). The relative abundance of dominant families was determined by the genescloud tools, and the Pathogen Detection database in NCBI and literature statistics were also used to identify pathogenic microorganisms and arbuscular mycorrhizal fungi (AMF), while metabolic functionality (PICRUSt) was done through the KEEG database.

## 3 Results

### 3.1 Soil physiochemical properties and toxic elements

**Figure 1:**
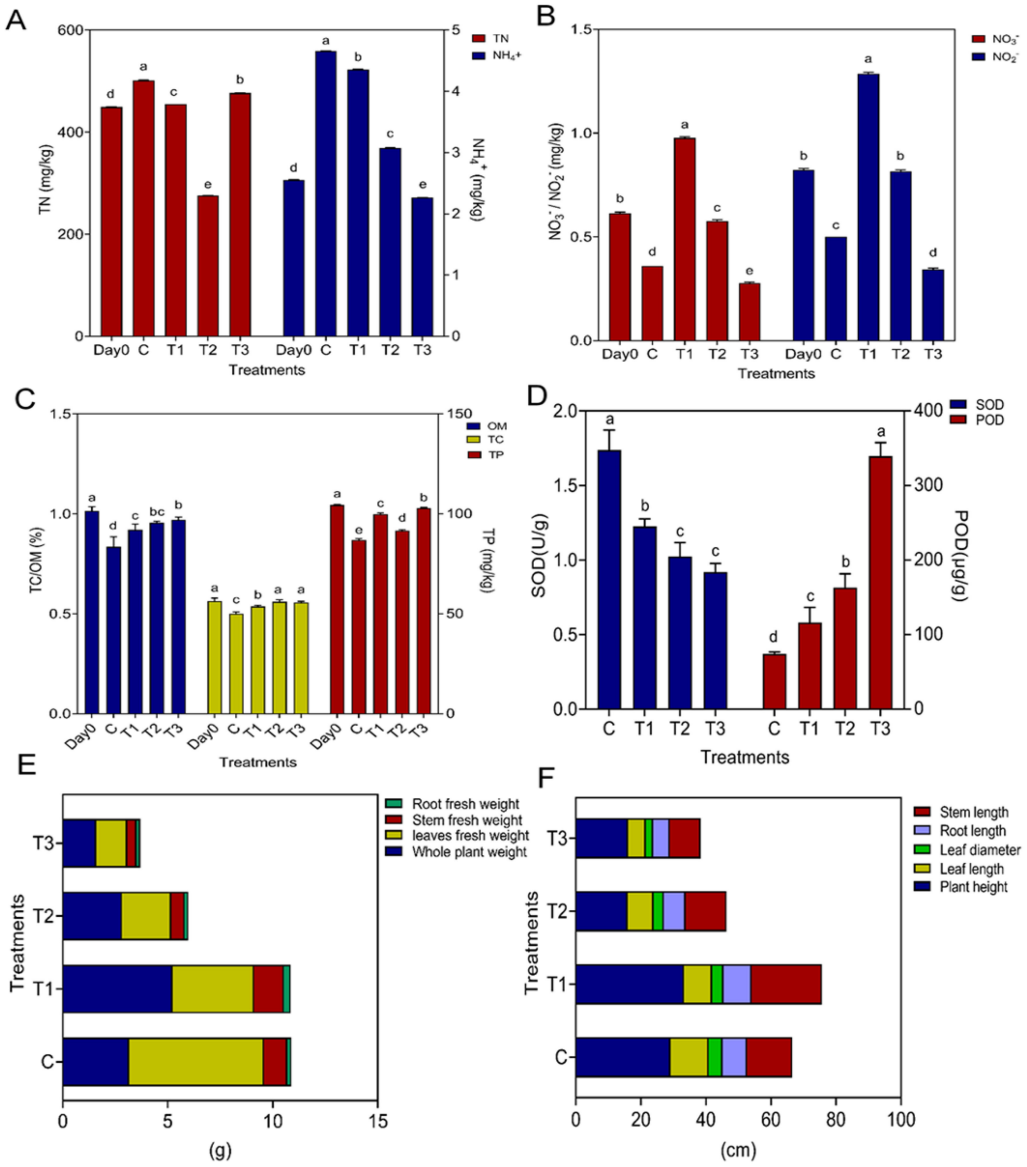
Plant physical, biochemical parameters and soil chemical properties. Figure **(A)** represents soil total nitrogen and ammonia; Figure **(B)** represents nitrate and nitrite; Figure **(C)** represents total phosphorus, total carbon and organic matter; Figure **(D)** represents SOD and POD activity of plant leaves; Figure **(E)** represents root fresh weight, stem fresh weight, leaves fresh weight, whole plant weight; Figure **(F)** represents stem length, root length, leaf diameter, leaf length, plant height; while C, control; T1, PS 106 µm; T2 , 50 µm, T3 = 13 µm.

The mean pH values in the heavy metal-enriched and contaminated with PS-MP soil differed significantly. The highest pH value (7.79) was recorded in T3, while the lowest pH (7.37) was recorded in day 0 treatments (**Supplementary Figure S2A**, **Supplementary Table S1**). The mean EC value in the T1 treatment (952 μS cm− 1) was significantly higher than in the other treatments (**Supplementary Figure S2B**, **Supplementary Table S1**). Soil organic matter (SOM), total phosphorus (TP), and total carbon (TC) were higher in treatment Day 0 (1.0100 mg/kg, 104.4200 mg/kg, and.5633 mg/kg). In contrast, the lowest values were recorded in treatment C (0.8433 mg/kg, 87.0067 mg/kg, and 0.5033 mg/kg, respectively) (**Figure 1C**, **Supplementary Table S1**).

The highest average content of total nitrogen (TN) and total ammonium nitrogen was observed in the control treatment (501.6667 mg/kg) and (4.6567 mg/kg), respectively, while total nitrate and total nitrite were in the T1 treatment (1.2876 mg/kg) and (0.9767 mg/kg), respectively. However, the lowest concentrations of total nitrogen, total ammonium nitrogen, nitrate, and nitrite were observed in the T3 treatment (**Supplementary Table S1**, **Figures 1A, B**). The concentration of the heavy metals Pb, Zn, and As was greater in the T1 treatment and Cd in the T3 treatment, while the concentration of Cu was higher in C, followed by T1. However, there was no significant difference between control and T1 (**Supplementary Figure S3**).

### 3.2 Plant physiochemical parameters

There were large differences in lettuce physiochemical attributes among different treatments due to HMs and Ps-MPs (**Figure 1**, **Supplementary Table S2**). The smallest PS-MPs treatment T3 significantly (*p* ≤ 0.05) reduced the growth characteristics of lettuce, such as whole plant weight, leaf diameter, length of stem, leaves, and root, fresh weight of leaves and stem, as well as their dry weight and root dry weight (**Figures 1E, F**, **Supplementary Figure S2C**, **Supplementary Table S2**).

The most decline in plant height and root fresh weight was recorded in T2 (15.9000 cm) and (0.1800 cm), respectively, while the lowest electrolytic leakage (32.2200) was observed in Control. There was no significant difference between treatment T2 and T3 in plant height and root fresh weight. RWC revealed a high concentration in C, while there was no significant difference among all treatments for electrolytic leakage (ELL) (**Supplementary Table S2**, **Supplementary Figure S2B**, **Figures 1E, F**).

#### 3.2.1 Enzymatic activity

In the case of SOD, more elevation (1.7382) was observed in the control as compared to the other treatments, while the lowest was recorded in (0.9197) T3, but there was no significant difference between T3 and T2. POD activity was completely opposite the SOD activity, as here the highest value was recorded in T3 (339.4667), while the more declined one (74.1333) was noted in Control (**Figure 1D**). The collected data and statistical analysis show that the HMs and PS-MP harshly impacted lettuce.

### 3.3 Effect of MPs and HMs on the soil microbial diversity

#### 3.3.1 Diversity of bacterial community

**Figure 2:**
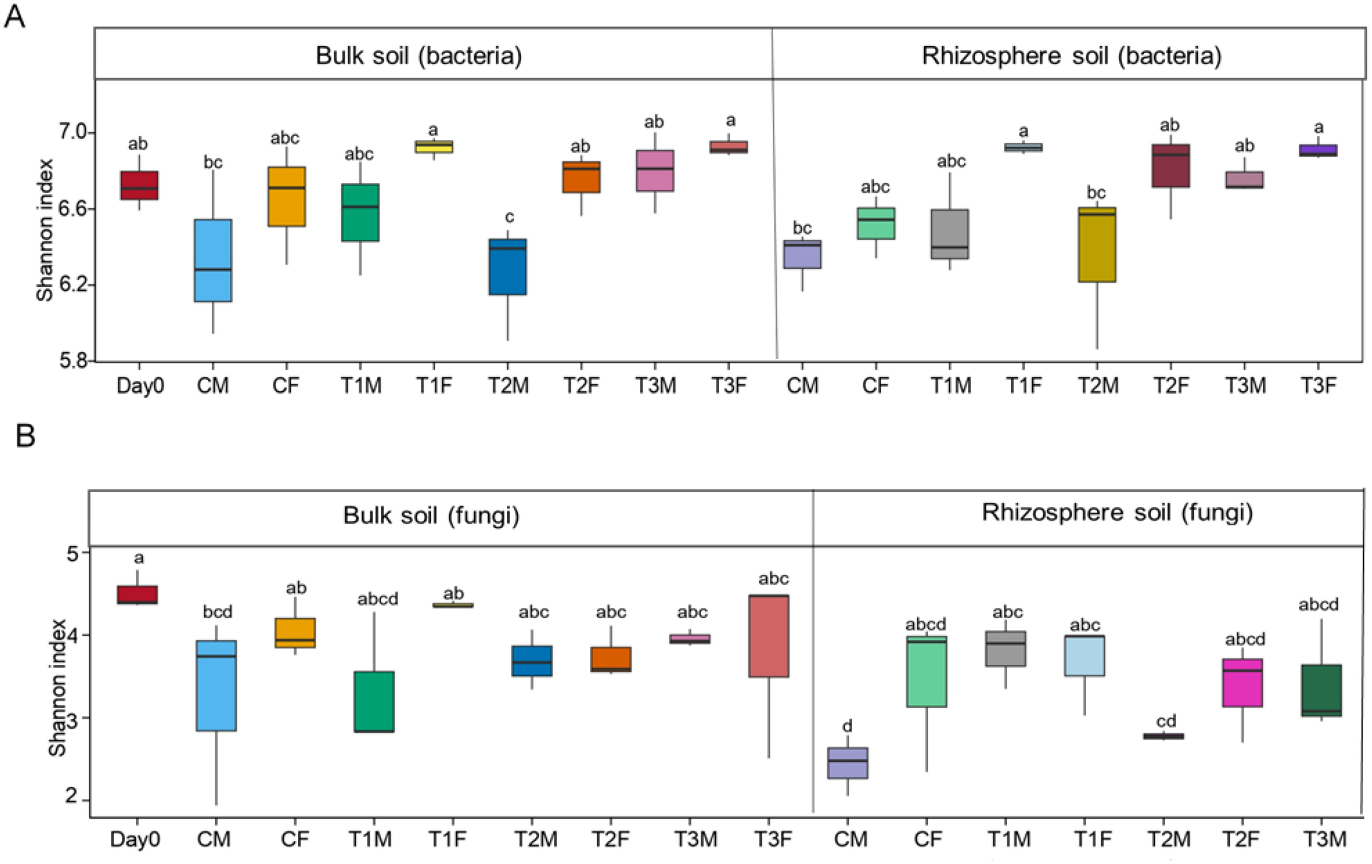
Alpha diversity of soil bacterial and fungal community. Figure **(A)** represents the Shannon index of bulk and rhizosphere soil of the bacterial community. Figure **(B)** represents the Shannon index of bulk and rhizosphere soil of the fungal community.; while M, middle sampling point; F, final sampling point; C, control; T1, PS 106 µm; T2 , 50 µm; T3 , 13 µm.

Overall treatment with smaller microplastic at the final and middle points shows higher *α*-diversity than other treatments in the soil bacterial community (Chao1, Shannon indices, observed species) (**Figure 2A**; **Supplementary Figure S4**). In comparison to the control, bulk soil bacteria revealed a significant difference at day zero, the middle, and the final points. At the same time, the control rhizosphere soil bacteria also showed a significant difference from other treatments except T1M. All treatments at the middle and final points differed from the control in bulk and rhizosphere soil, while in the comparison of rhizosphere and bulk soil, only T2M was significantly different. Overall, the highest *α*-diversity (6.9307) and lowest (6.2626) were denoted in T3BF and T2BM, respectively (ANOVA, Duncan test, Shannon index = 0.005). Overall, rhizosphere diversity and diversity at the final point were higher than the bulk and middle points (**Figure 2A**, **Supplementary Figure S4**).

The Chao1 index in bulk soil revealed a significant difference between the control and T2M and T2F. In contrast, an insignificant difference was observed between day zero, C, and T1 at the middle and final points. In rhizosphere soil, control revealed a significant difference among all the treatments, while there was no significant difference among T1, T2, and T3 at both the middle and final points. Furthermore, T1M, T1F, and T2F insignificantly differed when comparing rhizosphere and bulk soil. The highest *α*-diversity (4366.3044) and lowest (3391.5035) were denoted in T3BF and T2BM, respectively (**Figure 2A**).

#### 3.3.2 Diversity of fungal community

Fungal diversity was higher at the final point and in bulk soil as in contrast to the middle point and rhizosphere soil, respectively, in Shannon, Observed species, and Chao1 indices. (**Figure 2B**, **Supplementary Figure S5**). In the bulk soil, all the treatments revealed significant differences from the control at both the middle and final points, except T1F. In the rhizosphere soil at the middle point, all the treatments revealed significant differences, while at the final point, T2F and T3M revealed insignificant differences from CF. In the comparison of bulk and rhizosphere soil, all the treatments showed significant differences except T3F. Overall, the highest *α*-diversity (4.5146) and lowest (2.4415) were denoted in day 0 and CRM, respectively (ANOVA, Duncan test, Shannon index = 0.030) (**Figure 2B**).

The Chao1 index in bulk soil revealed a significant difference at the middle point, while at the final point, the control showed a significant difference from T1F. In the rhizosphere soil, all the treatments differed significantly from the control at the middle point, while at the final point, T2M and T3M revealed insignificant differences. In comparison with the rhizosphere and bulk soil, T2M revealed an insignificant difference. The highest *α*-diversity (700.2727) and lowest (458.0311) were denoted in T1BF and T1BM, respectively (ANOVA, Duncan test: Shannon index = 0.039) (**Figure 2B**, **Supplementary Figure S5**).

### 3.4 Effect of MPs and HMs on the soil microbial composition and its metabolic functions

#### 3.4.1 Microbial community composition

**Figure 3:**
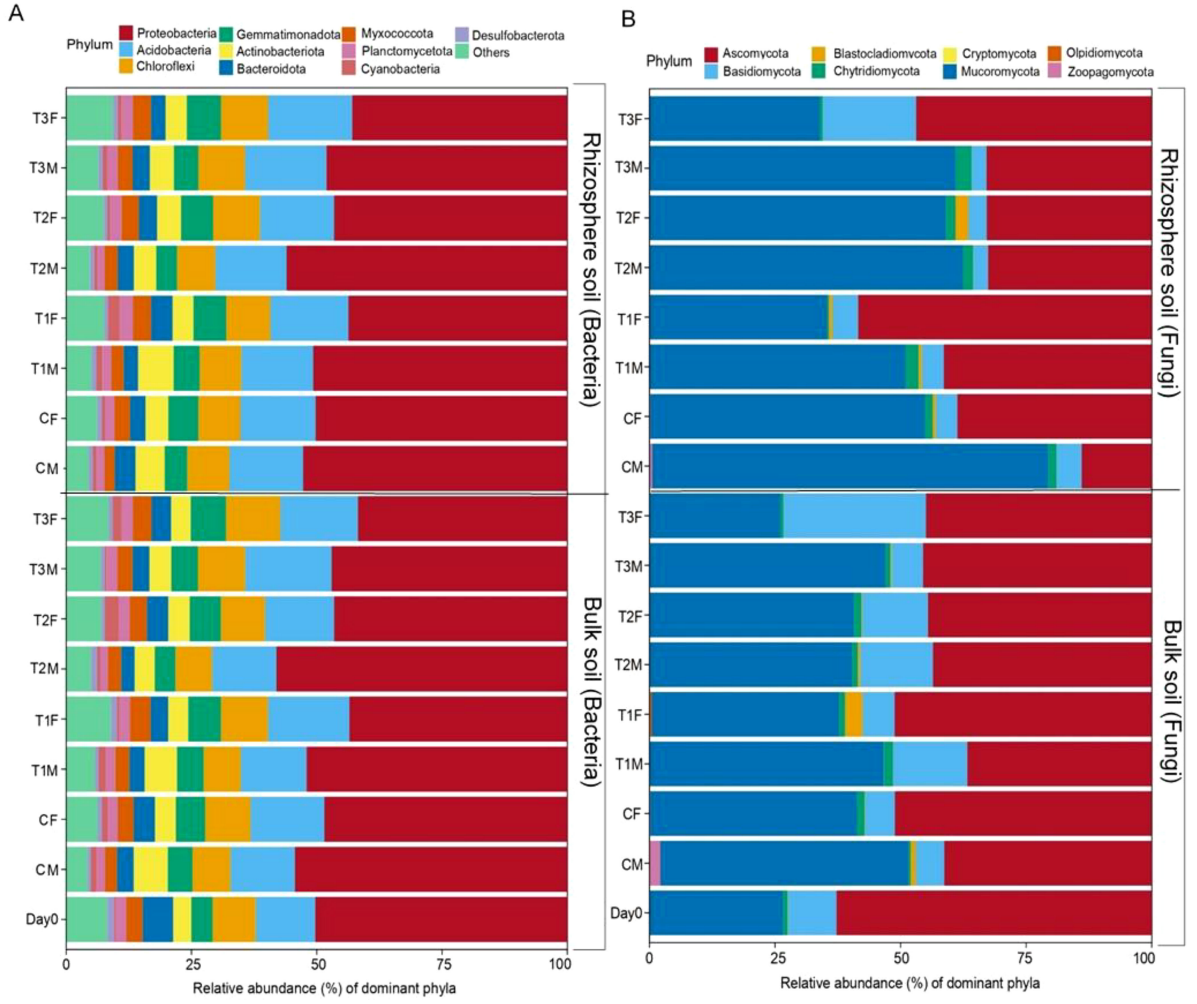
Beta diversity of soil fungal and bacterial community. Figure **(A)** represents the relative abundance of bacterial dominant phyla of bulk and rhizosphere soil. Figure **(B)** represents the relative abundance of fungal dominant phyla of bulk and rhizosphere soil; while M= middle sampling point, F=final sampling point, C, control; T1, PS 106 µm; T2 , 50 µm; T3 , 13 µm.

The most abundant bacteria phylum in our data were Proteobacteria (49.01%), Acidobacteriota (14.70%), Chloroflexi (8.68%), Gemmatimonadota (5.52%), and Actinobacteria (4.80%). The lowest abundance of Proteobacteria was found in T3BF (41.81%), followed by T3RF (42.95%), while the highest abundance of Acidobacteriota was observed in T3BM (17.26%) and T3RF (16.76%) (**Figure 3A**).

The most dominant fungal phylum in our study was Mucoromycota (46.52106%), Ascomycota (42.3413%), Basidiomycota (8.897272%), and Chytridiomycota (1.421189%), while Cryptomycota was the phylum with the lowest abundance and was only found in the bulk soil at the middle point in T1 and T3. The highest abundance of Mucoromycota was recorded in rhizosphere soil at the middle point (78.85%) (62.37%) in control and T2, respectively, while in Ascomycota, it was recorded on Day 0 (62.79%) and T1RF (58.46%). (**Figure 3B**).

#### 3.4.2 Pathogenicity

**Figure 4:**
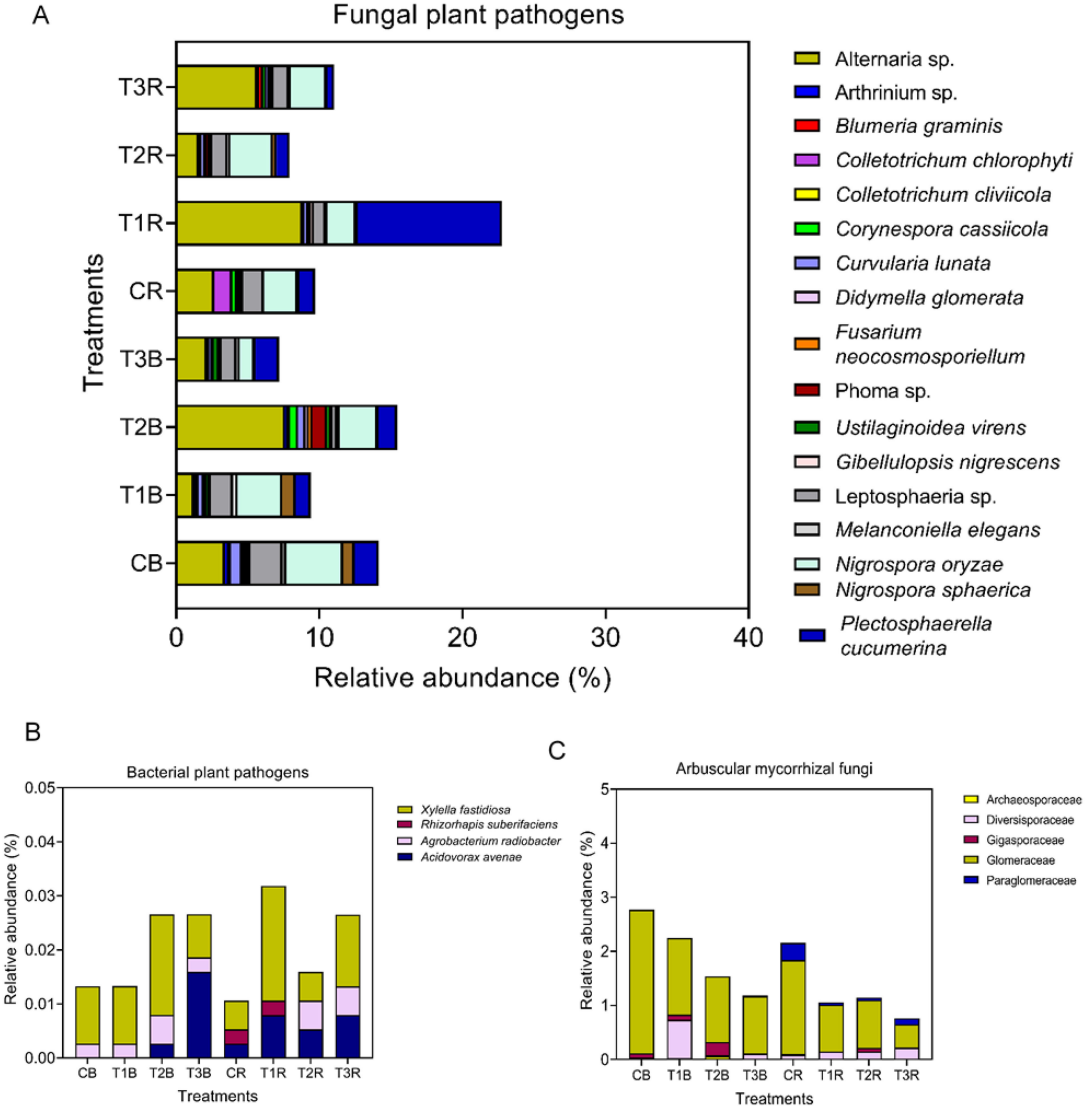
Bacterial and fungal plant pathogens and arbuscular mycorrhizal fungi (AMF). Figure **(A)** represents relative abundance of fungal plant pathogen species in bulk and rhizosphere soil. Figure **(B)** represents relative abundance of bacterial plant pathogen species in bulk and rhizosphere soil and Figure **(C)** represents relative abundance of arbuscular mycorrhizal fungi in bulk and rhizosphere soil at family level; while C, control; T1, PS 106 µm; T2 , 50 µm; T3 , 13 µm; B, bulk soil and R, rhizosphere soil.

Our study revealed that changes also applies to the microbial pathogens, and mostly rhizosphere soil and small PS-MPs treatments were more enriched in microbial pathogens (**Figures 4A, B**). A total of 10 fungal plant pathogens were observed across all the treatments at the species level. Alternaria sp. was the most abundant pathogen species, followed by *Nigrospora oryzae* and *Plectrosphaerella cucumerina*. In the bulk soil, the highest abundance of *Nigrospora oryzae*, Leptosphaeria sp., and *Curvularia lunata was* in CB, and *Ustilaginoidea virens* were present in T3B. As for the rhizosphere soil, Alternaria sp. and *Plectosphaerella cucumerian* revealed more abundance in T3 and T2, respectively. Fungal pathogenic species *Didymella glomera*, *Blumeria graminis*, and *Corynespora cassiicola* were found in T2 and T3 in rhizosphere soil, while Phoma sp. was present in T2 and T3 in both rhizosphere and bulk. Surprisingly, *Collectrotrichum chlorophyte* was also found in rhizosphere soil but in control treatment (**Figure 4A**).

Four bacterial plant pathogen species were present; overall, bacterial plant pathogen species also revealed more abundance in rhizosphere soil than in bulk soil. *Xylella fastidiosa* and *Rhizorphapis suberifaciens* were present in high abundance in T1R, while *Acidovorax avenae was* in T3B. We found *Rhizorphapis suberifaciens* in rhizosphere soil only in C and T1, but there was no significant difference among both (**Figure 4B**).

#### 3.4.3 AMF

Five arbuscular mycorrhizal fungi were identified at the family level. The most dominant family was Glomeraceae, followed by Diversisporaceae, Gigasporaceae, Paraglomeraceae, and Archeosporaceae. Glomeraceae (2.65%) and Paraglomeraceae (0.32%) were more abundant in the CB and CR, respectively. The Glomeraceae family revealed a decline in T3 in the rhizosphere and bulk soil. Higher abundance of Diversisporaceae and Gigasporaceae in T1B and T2B, respectively. Archeaosporaceae were only present in T2 (R+B) and T1R (**Figure 4C**).

#### 3.4.4 Predicted metabolic functions

**Figure 5:**
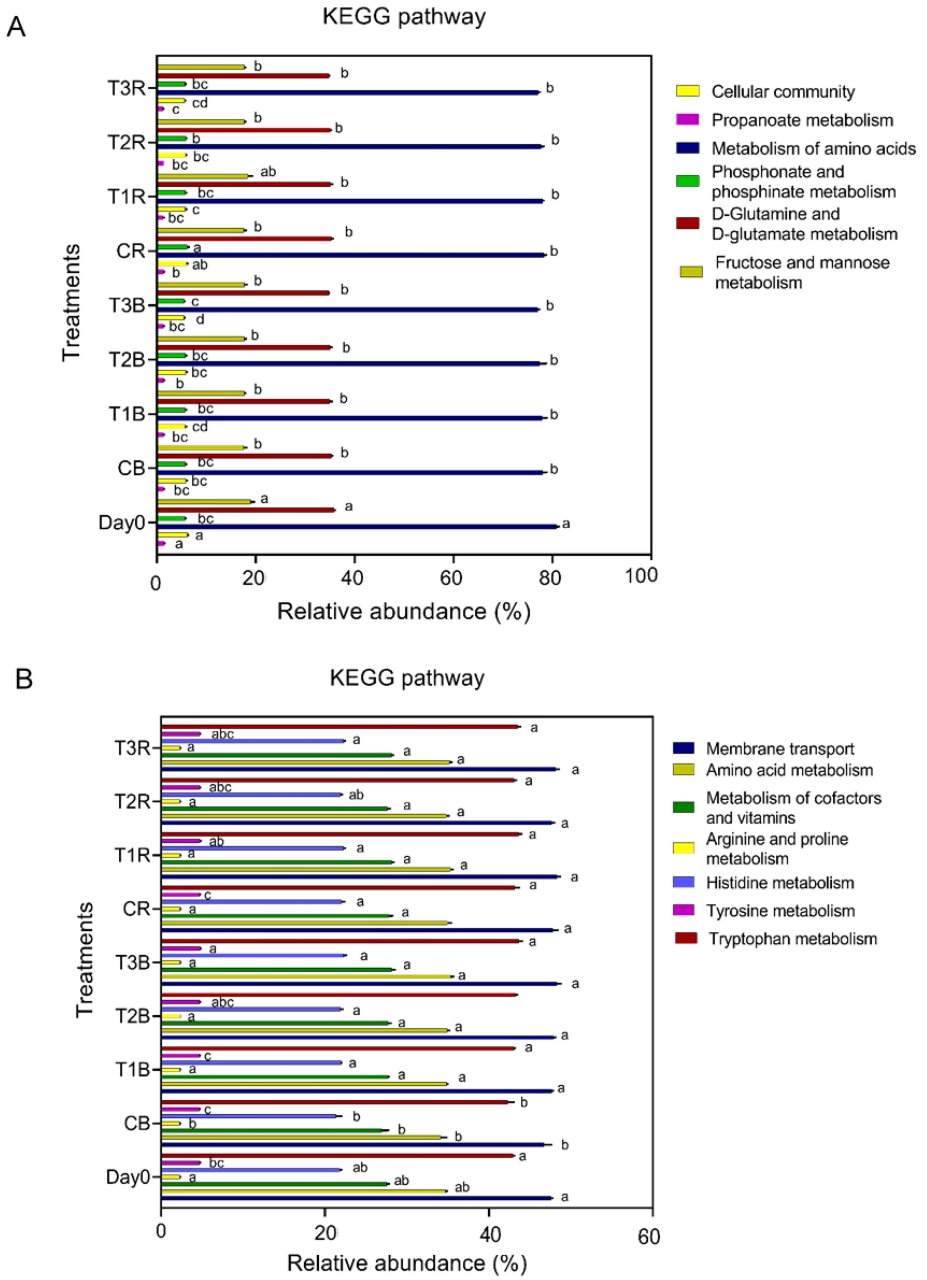
KEGG Pathway. Figure **(A)** represents KEGG pathway at bacterial level. Figure **(B)** represents KEGG pathway at fungal level. While C, control; T1, PS 106 µm; T2 , 50 µm; T3 , 13 µm; B, bulk soil and R, rhizosphere soil.

PICRUSt analysis investigated the functional genes associated with soil microbes regarding metabolism, cellular community, and environmental availability (**Figure 5**). For the fungal predicted metabolic functions, control of the bulk soil (CB) revealed significant differences with all other treatments; however, in amino acid metabolism, arginine and proline metabolism, membrane transport, and tryptophan, there were insignificant differences among different treatments. While there was no significant difference for the tyrosine between CB, CR, and T1B and histidine metabolism in CB and Day 0, the metabolism of cofactors and vitamins at Day Zero also differs from CB and other treatments (**Figure 5B**).

For the all-given bacteria predicted metabolic functions, day 0 differed significantly from the rest of the treatments except for phosphonate and phosphinate metabolism; however, control of rhizosphere soil also revealed significant differences in cellular processes, phosphonate and phosphinate metabolism from all the treatments, while there were insignificant differences for propanoate metabolism in CR and T2B. T3R and T3B also differed significantly from all other treatments in propanoate metabolism and phosphonate and phosphinate metabolism (**Figure 5A**).

### 3.5 Relationship between soil physicochemical variables and microbial community

**Figure 6:**
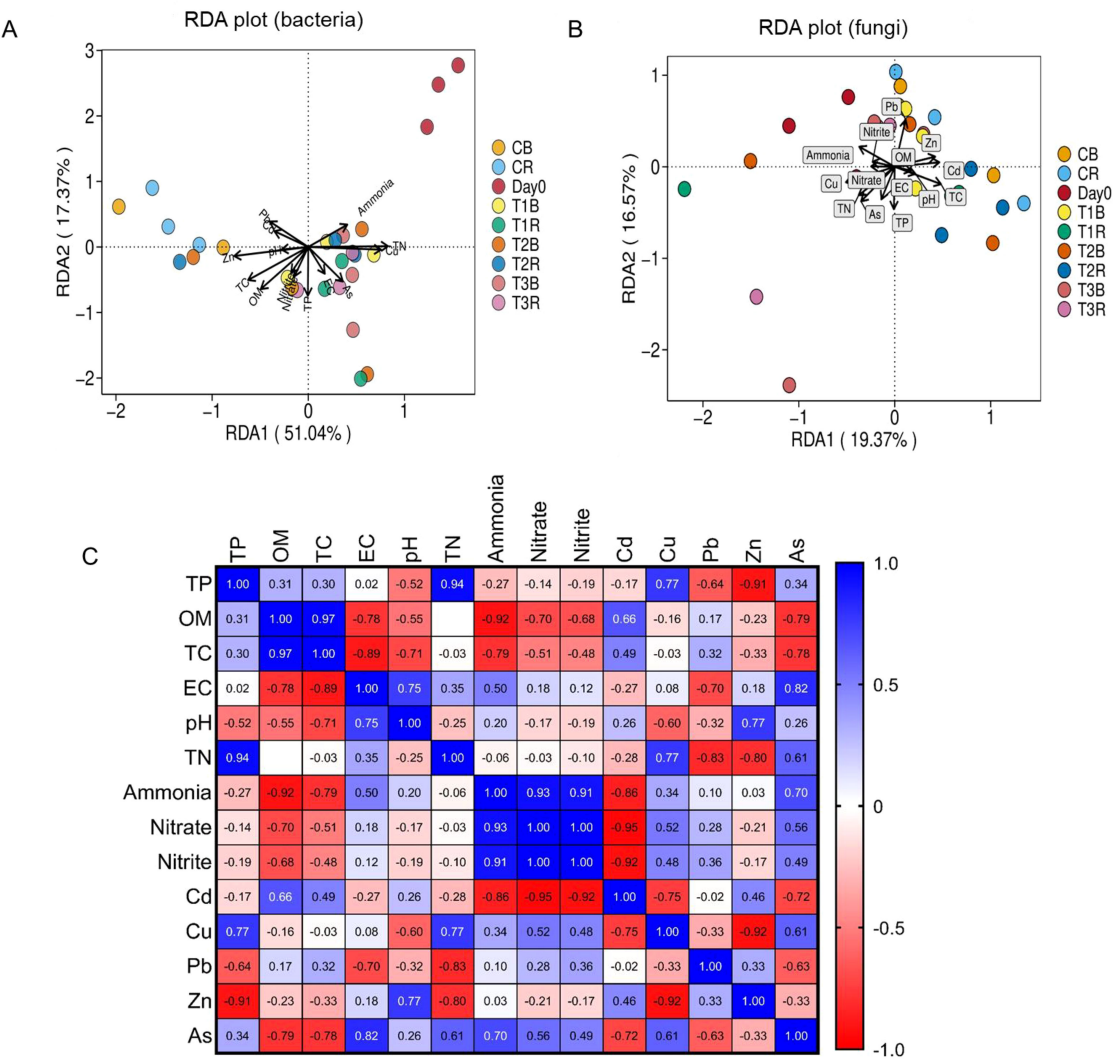
RDA analysis of the effect of environmental factors on microbial composition. Figure **(A)** represents RDA plot for composition. Figure **(B)** represents RDA plot for fungal composition. Figure **(C)** represents Matrix test with a Pairwise comparisons of soil indicators are shown, with a color gradient denoting Pearson’s correlation coefficient; while C, control; T1, PS 106 µm; T2 , 50 µm; T3 , 13 µm; B, bulk soil and R, rhizosphere soil.

The association between environmental factors and microbial communities was assessed using redundancy analysis (RDA) (**Figures 6A, B**). Matrix test with pairwise comparisons of soil indicators, with a color gradient denoting Pearson’s correlation coefficient (**Figure 6C**). The first two RDA dimensions showed a 68.41% variation in bacterial communities. Environmental factors such as ammonia, TN, Cu, EC, and As were positively correlated with RDA1. Furthermore, more abundance was also present at RDA1 (**Figure 6A**). The first two RDA dimensions explained 35.91% of the variation in fungal communities. Environmental factors Zn, Cd, TC, pH, EC, TP, and OM were positively correlated with RDA1, and more abundance was also present in RDA1 (**Figure 6B**).

## 4 Discussion

### 4.1 Micro plastic addition affected soil

Microplastics Changed the soil environment, and their addition altered soil physiochemical parameters in this study. Until now, combining soil with contamination of heavy metals and microplastics has gained more attraction, leading to changes in the soil properties. The metal concentrations were not considered constant because the soil is not a uniform medium (Wang et al., 2020; Dong et al., 2022; Kim et al., 2017; Dong et al., 2021; Meng et al., 2023; Hansel et al., 2001; Cao et al., 2021; Zhao et al., 2023). The interaction mechanisms of MPs and NPs at the surface of soil-roots are complex. The rhizosphere processes are also induced by changes in soil pH, organic matter, mineral composition, and microbial communities (Ameer et al., 2022; Bombino et al., 2023; Liu Y. et al., 2021; Zhu F. et al., 2022). Previous studies reported that changes in soil factors may further cause opposite or negative effects on MPs translocation and bioavailability and shape and type of MPs also effect the soil physical properties (Qi et al., 2022; Wang et al., 2022; Chen et al., 2022c).

Soil pH is among the key soil properties determining the mobility of nutrients (Marschner and Rengel, 2012) and heavy metals (Zeng et al., 2011). In our results, MPs caused a higher soil pH than the corresponding treatment, which received no microplastics (**Supplementary Table S1**, **Supplementary Figure S2A**). This was contrary to the widely accepted fact that heavy metal pollution in the soil may lead to reduction of the soil pH (Sun et al., 2023). The possible reason may be that smaller microplastics have a larger surface area-to-volume ratio compared to larger microplastics. This increased surface area may allow for more interactions with soil components, including the adsorption of acidic substances or the release of basic compounds which results in a relative increase in pH. Our results confirmed the interactive effects between MPs and heavy metals. MPs may alter the environmental behaviors of heavy metals in soil, such as dissolution, precipitation, hydrolysis, and sorption, and further their impacts on soil pH. The addition of MPs influenced soil pH; however, the effects depended on MPs size. Soil pH increased gradually with decreasing PS-MPs size, regardless of HMs. This fact confirms that the coexistence of HMs can change MPs’ impacts on soil properties. Previously, after two months of soil culture, both low-density PE and biodegradable plastic mulch films with a size of 50 mm to 1 mm induced an increase in soil pH, which further increased after another two-month culture (Qi et al., 2020). We found contrasting trends, probably due to their different biodegradation characteristics. We found no decrease but a substantial increase in soil pH.

Organic matter is absorbable and can adsorb Cu in the soil. Therefore, soil organic matter was negatively correlated with soil Cu content. In their study, Qi et al. (2018) mentioned that organic matter can adsorb heavy metal elements in the soil. Lin et al. (2021) believed that soil organic matter plays a prominent role in the adsorption of heavy metals and geo-biochemical processes of environmental contaminants. These studies also verify the conjecture in this research that organic matter can adsorb Cu in soil, which makes the soil organic matter negatively correlated with the soil Cu content.

There are still many unknowns in active research on the relationship between microplastics and soil nutrients. Research is ongoing to understand the mechanisms and implications of nanoplastic interactions in soil ecosystems. Microplastics could potentially influence plants’ uptake of nutrients. In our studies, TP and TC were in greater quantity on day 0, while they were lowest in the control treatment, so we can assume that in the control treatment, they were lowest due to the uptake by the plant, which showed an increasing trend with the decreasing size of microplastics. Thus, Serrano-Ruiz et al. (2021) and van den Berg et al. (2020) also reported that nutrients in soil may be affected by MPs and plant uptake of nutrients and water because of their consistent transport pathways.

### 4.2 PS-MPs effect on lettuce physiochemical attributes

To date, very few studies have been conducted on MPs impacts on the growth of higher plants, and various influences have been observed (Xu et al., 2023). Studies reported that microplastics have been found to postpone seed germination, decreasing water and nutrients absorption, and damaging antioxidant defense systems (Chen et al., 2023b; Azeem et al., 2023; Bosker et al., 2019), reduce plant growth (Qi et al., 2018, 2020), and induce genotoxicity and ecotoxicity in plants (Zhang et al., 2023c).

Previous studies reported that owing to the large specific surface area of MPs, various contaminants or nanomaterials, such as engineered nanomaterials, polychlorinated biphenyls, polycyclic aromatic hydrocarbons, and toxic heavy metals, can be adsorbed on their surfaces and transported upward through the xylem to the upper parts of the plant (Kah et al., 2018; Bradney et al., 2019; Deng et al., 2021; Chen et al., 2022b). Zhang et al. (2020b) reported that particle size is another important factor affecting the transport of MPs and plant physiology. Dong et al. (2021) and Liu et al. (2022) also reported that nanoscale (1–1 000 nm) and even micron-scale (sub-1 μm) plastics have been shown to be absorbed by roots and transported to the aboveground, and micron-scale plastics accumulate mainly in the roots. Our studies also showed the remarkable effect of the contaminants on the plant indicators. Our findings revealed a noteworthy decrease in the whole plant weight, leaf diameter, length, dry and fresh weight, stem length, dry and fresh weight, root length, and dry weight in the T3 treatment with the smallest size of PS-MPs. The lowest root fresh weight and plant height were observed at their lowest in the T2 treatment, but there was no significant difference with T3. Our results revealed the lowest concentration of As, Cu, Zn, and Pb in the soil at harvest time. These results confirmed that MPs phytotoxicity highly depends on MPs size, i.e., T1 showed no obvious phytotoxicity, while T2 and T3 exhibited high phytotoxicity and proved that microplastics enter the plant parts and block the pathway for nutrient uptake, thus reduce their growth. Our results revealed that MPs help in the transportation of these metals to the plant parts and reduce their growth. Our results are also similar to the findings of Wang et al. (2020). (Lian et al., 2020) also proposed that most of the MPs are present only in the vascular system due to the particle size and weight, while MPs with smaller particle sizes are more easily transported upward through the xylem. Therefore, we believe that the range of MP particles in soil can be an important factor affecting the agronomic traits of higher plants.

Surprisingly, Cd findings were contrary to our expectations because the smallest MPs were supposed to decrease the availability of Cd concentrations in the soil while transporting to plant parts and higher accumulation in plants. However, Cd revealed a strange trend, decreasing its concentrations in T1 and then increasing in T3, followed by T2. We suggest that there must be other factors influencing Cd uptake by plants and Cd dissolution with MPs in lettuce. More focus is needed on the Cd pathway and adsorption in lettuce under the effect of MPs.

Plant physiology in terms of relative water content (RWC) was significantly reduced in contaminants when compared to the control. This parameter decrease might be due to membrane damage (ELL). Our results revealed high membrane leakage in T3 and indicated membrane damage due to lettuce exposure to stress. These findings are supported by the study of (Chen et al., 2022a; Wang et al., 2024; Yang et al., 2024), who proposed that heavy metals stress produced ROS that caused oxidative stress and increased membrane leakage which leads to the production of MDA. Our findings are also similar to previous research that reported reduced RWC and increased ELL and MDA contents in plants due to biomembrane damage caused by heavy metal stress (Chen et al., 2022a; Yang et al., 2024).

The level of superoxide dismutase (SOD) gradually decreased with the decrease in MP size in our study, while POD showed a completely opposite interaction. Our results proved that lettuce has encountered various environmental stressors, such as drought due to the hydrophobic nature of MPs and heavy metals, which makes the plant more vulnerable to these stress factors, which potentially impact the plant’s ability to defend itself against oxidative stress, and this phenomenon is well established and also explained in the prior studies (Chen et al., 2022a; Wang et al., 2024; Yang et al., 2024). However, these findings are contrary to the findings of Ahmad et al. (2018). We suggest that this decrease in SOD activity might be a compromised antioxidant defense system against superoxide radicals. This could be due to various factors such as genetic factors, more environmental stress, or a specific physiological condition in the lettuce. Increased POD activity may suggest a response to elevated levels of hydrogen peroxide, which can result from the dismutation of superoxide radicals in the absence of sufficient SOD.

### 4.3 Microbial diversity, compositions, pathogenicity and metabolic functions

#### 4.3.1 Microbial diversity and composition

Zhu J. et al. (2022) reported that MPs in soil provide a nutrient source for microorganisms and become a unique microbial habitat. Different types of microplastic reduced the diversity of the rhizosphere soil microbes to varying degrees. (Rong et al., 2021) found that different concentrations of polyethylene-microplastics had a slight effect on soil bacterial diversity; however, there were differences at the genus level. Yang et al. (2021) found that MPs with different particle sizes and concentrations altered the physicochemical indicators of the soil and the growth of Chinese cabbage. Rillig et al. (2024); Yang et al. (2018) and Amaral-Zettler et al. (2020) also stated that in addition to contamination by MPs, various studies have shown that changes in soil microbiology can reduce and degrade persistent pollutants, such as HMs and tetracyclines. However, there are few reports on how different particle sizes and concentrations of MPs affect plant rhizosphere bacteria and very few studies on the combination of MPs, HMs, and microbes and most studies are focused only on aquatic ecosystems. Qi et al. (2020) indicated that biodegradable MPs induced stronger impacts on bacterial community composition in the wheat rhizosphere. However, there is a lack of work on the non-biodegradable MPs.

Our study is the first, which is based on the combination of PS-MPs and HMs and their effects on higher plant species (*Lactuca sativa)*, AMF, bacterial and fungal pathogens, microbial diversity, and composition at different points, including day zero, middle point, and final point, with both rhizosphere and bulk soil.

Soil bacterial analysis showed that the treatment with small MPs at the final point led to a significant increase, followed by the same at the middle point in the Shannon, observed species, and Chao 1 indices, while overall rhizosphere diversity was greater than bulk diversity (**Figure 2A**, **Supplementary Figure S4**). Therefore, our study confirmed that microplastic size, different time zones, and spheres play an important role in the diversity of bacteria. Particle size could be the cause of this. In the soil, MPs with various particle sizes can be found at varying ranges and depths. Large-sized MPs are more likely to be found on the soil’s surface, and the contamination range of MPs depends on their concentration. Smaller-sized MPs may seep into deeper soils that receive rainfall. This is also supported by the study of Vick-Majors et al. (2014) who revealed that the depth and extent of MP particles in the soil might be important factors affecting microbial diversity indicators.

Soil fungal analysis also showed more diversity at the final point than at the middle point, while overall diversity in bulk soil was higher than the rhizosphere soil in Shannon, Observed species, and Chao1 indices (**Figure 2B**, **Supplementary Figure S5**). This may be due to the higher abundance of AMF Gigasporaceae and Glomeraceae in the bulk soil. These fungi contribute to the overall fungal diversity in ecosystems, and their presence can influence the composition and dynamics. (Zhang Z. et al., 2019) also stated that microplastics are “special microbial accumulators.” In the comparison of treatments, fungal diversity was higher on day 0, T1, and de Souza MaChado et al. (2019) stated that however, to date, only one publication has reported that mycorrhizal symbiosis was affected by MPs. Previous studies also reported that Soil microorganisms are the basis of Earth’s biosphere and play an irreplaceable role in nutrient cycling and organic matter decomposition (Vicuña and González, 2021; Zhang, 2023). However, differences in the responses of different microbial taxa to environmental factors cause different adaptations to environmental gradients (Chase et al., 2021). Rong et al. (2021) found that different concentrations of PE-MPs had a slight effect on soil bacterial diversity, while Rillig (2012) illustrated that MPs directly affect soil quality and fertility by altering soil nutrient cycling and microbial communities. Qi et al. (2022) did not find any significant difference in the composition, diversity, and structure of bacteria and fungi.

In our study, bacteria revealed higher growth rates of different phyla compared with fungi, which is consistent with the studies of Loeuille et al. (2017) and Fukami et al. (2010). This may be due to the fungi’s limited dispersal ability through the growth of their mycelium, which can be affected by environmental factors.

The most abundant bacterial phyla in our study were Proteobacteria, Acidobacteriota, Chloroflexi, Gemmatimonadota, and Actinobacteriota. The lowest abundance of Proteobacteria was found in T3BF, followed by T3RF, while surprisingly, the highest abundance of Acidobacteriota was also observed in T3BM and T3RF (**Figure 3A**). So, our results suggest that Proteobacteria were affected by MPs, but Acidobacteriota were abided by the contamination of PS-MPs. It can be suggested that Acidobacteriota can exhibit tolerance to the elevation of HMs by different mechanisms, including efflux pumps, metal-binding proteins, and other adaptations; it may adhere to the surface of MPs, which leads to change in its microbial community dynamics. Another reason could also be that there may be some species within the Acidobacteriota phylum that exhibit specific adaptations that allow them to colonize and thrive in various soil-contaminated environments. However, there are very few reports about the bacterial composition in the combination of PS-MPs and HMs.

The most dominant fungal phylum in our study was Mucoromycota, Ascomycota, Basidiomycota, and Chytridiomycota (**Figure 3B**). In our research, Cryptomycota was the phylum with the lowest abundance and was only found in the bulk soil in T1 and T3; this also supports the higher abundance of fungal diversity in the bulk soil, as was the case with the Ascomycota, which was recorded in Day 0 and T1 treatment at the final point. The highest abundance of Mucoromycota was recorded in rhizosphere soil, at the middle point in Control and T2, respectively (**Figure 3B**). Hence, our results illustrate that the top two abundant phyla were present on Day 0, C, T1, and T2, respectively, which means the smaller size of PS-MPs decreased their abundance.

#### 4.3.2 AMF

In our studies, the AMF community reduced with the decrease in MPs size. AMF abundance was higher in C, followed by T1. T1 and C showed more abundance in the Diversisporacceae and Paraglomeraceae, respectively, but they decreased with the decrease in MPs size (**Figure 4C**). It indicated that the smallest MPs have adverse effects on the AMF community, especially the rhizosphere AMF community, which is very dangerous. Previous studies revealed that AMF is considered among the most ubiquitous probiotic microorganisms that form symbioses with a vast majority of higher plants in various environments (Noceto et al., 2021; Wang J. et al., 2019; Shi et al., 2020). Alguacil et al. (2022) also proposed a significant reduction of AMF family richness in all environmental manipulation treatments as compared to the control treatment, and members of the Gigasporaceae and Diversisporaceae families appeared to be highly vulnerable to intensification of stress as their abundances decreased. A myriad of studies has confirmed that heavy metal pollution negatively affects AMF abundance and diversity (Wang J. et al., 2019). In this context, MPs may influence AMF community structure directly or indirectly by altering soil properties, including heavy metal bioavailability and toxicity (de Souza MaChado et al., 2018; Zhang et al., 2020a). Undoubtedly, there is a lack of evidence on the AMF response to MPs and coexisting heavy metals.

For the first time, our outcomes confirm that MPs alter AMF community structure and diversity depending on PS-MPs size. Before, only Wang et al. (2020) confirmed that MPs can change AMF community structure and diversity, depending on MPs type and dose, but Cd and PE did not statistically influence AMF diversity but substantially changed their community structure. In our study, MPs changed the abundance of AMF by reducing it with a decrease in MPs size. Numerous previous studies have stated highly conflicting findings on PE influences on the richness and diversity of soil bacterial communities, ranging from negative (Fei et al., 2020), insignificant (Huang et al., 2019), and positive (Ren et al., 2020). Our study is also supported by Xiang et al. (2023), who revealed that microorganisms undergo a range of physiological and metabolic changes in response to short-term MPs pollution-induced environmental stress in order to adapt and cope.

Certain microbial populations had a drop in abundance as a result of MPs pollution, suggesting that they are susceptible to the environmental stress that MPs pollution causes.

#### 4.3.3 Pathogenicity

Our study illustrates that the MPs changes not only diversity but also microbial pathogens (**Figures 4A, B**). (Qi et al., 2022) also demonstrate that the plastisphere may also act as a reservoir of fungal pathogens, and future research on the impact of plastic residues and the prevalence of soil-borne diseases would be crucial. Our results showed that mostly rhizosphere soil and small PS-MPs treatments were richer in bacterial and fungal pathogens. This may also be the reason behind the reduction in lettuce growth. Fungal pathogenic species *Didymella glomera*, *Blumeria graminis*, and *Corynespora cassiicola* were found in T2 and T3 in rhizosphere soil, while Phoma species were present in T2 and T3 in both rhizosphere and bulk soil. Surprisingly, *Collectrotrichum chlorophyte* was also found in rhizosphere soil, but in control treatment. We expected more bacterial pathogens, but our results were not surprising. In a previous study (Yu et al., 2018), it was also stated that the potential risks of food poisoning through lettuce consumption might be higher in some specific months. However, the proportion of pathogenic bacteria in lettuce was relatively low.

For bacterial pathogens, we found *Rhizorphapis suberifaciens* in rhizosphere soil. Hence, it proved that MPs can interfere with microbial pathogens. This can also be affected by the less AMF in the rhizosphere and the contamination of MPs. Other studies also stated that environmental contaminants can interfere with the microbial community. For some characteristics and functions, the microbiome of both soil and plants is extremely important (Berg et al., 2021; Liu et al., 2020b). Gregory et al. (2022) reported that one of the earth’s most dynamic interfaces is the rhizosphere, the small area that surrounds and affects plant roots. Qi et al. (2020) also stated that however, it has come to our attention that the bacterial community in the rhizosphere may be significantly impacted by the introduction of microplastics. Previous studies also reported *Phoma* spp. as a fungal pathogen of potatoes that causes gangrene (Steglińska et al., 2023); *Blumeria graminis* is among the top seven pathogens that cause powdery mildew (Savary et al., 2019); Rhizorphapis suberifacien**s** is the causing agent of corky root in *Lactuca sativa* (van Bruggen et al., 2015); Boucher and Wick (2004) reported *Pa*. *cucumerina* as the causal agent of Plectosporium Blight in zucchini and pumpkin and Mirtalebi et al. (2022) in melon however, our study is reporting it as a lettuce pathogen also. In previous studies, *C. chlorophyti* has been described as a pathogen in numerous herbaceous plant species and in important crop plants, especially legumes such as soybeans and tomatoes (Paniz -Mondolfi et al., 2021; Pandey et al., 2023).

#### 4.3.4 Metabolic pathways

We did a PICRUSt analysis to investigate the functional genes associated with soil microbes in terms of metabolism, cellular community, and environmental information processing (**Figure 5**). In our findings, all given bacteria predicted metabolic functions, but Day 0 differed significantly from the rest of the treatments except for phosphonate and phosphinate metabolism. However, control of rhizosphere soil also revealed significant differences in cellular processes, phosphonate and phosphinate metabolism from all the treatments, while there were insignificant differences for propanoate metabolism in CR and T2B. T3R and T3B also differed significantly from all other treatments in propanoate, phosphonate, and phosphinate metabolism (**Figure 5A**).

For the fungal predicted metabolic functions, control of the bulk soil (CB) revealed significant differences with all other treatments; however, in amino acid metabolism, arginine and proline metabolism, membrane transport, and tryptophan, there were insignificant differences among different treatments. No significant difference was found in the tyrosine in between CB, CR, and T1B, and for histidine metabolism in CB and Day 0. The metabolism of cofactors and vitamins on Day Zero also differs from CB and other treatments (**Figure 5B**).

Hence, it can be concluded that control and day 0 treatments differed from MPs-contaminated treatments in the majority of the metabolic functions, so it can be summarized that control and larger-sized MPs don’t vary as much in metabolic functionality. HMs also did not affect metabolic functions alone, which may be due to the presence of AMF species. There may be a reason that AMF abundance was higher in the absence of MPs, so it may have played a role in the metabolic functions and shown some resistance to the HMs. AM fungi, especially species of Glomeraceae, may exhibit a degree of tolerance to certain heavy metals, and these fungi may have mechanisms to tolerate or accumulate heavy metals in their hyphae without experiencing detrimental effects. While AM fungi may show some level of tolerance or adaptation to heavy metals, the impact of heavy metal contamination on mycorrhizal associations can have implications for changes in metabolic functions, plant health, and ecosystem functioning. However, there is no data available regarding this to date, but ongoing research continues to explore the intricate interactions between AM fungi, plants, and heavy metals in various environmental contexts. This can also be linked with the maximum presence of Cd in the MPs treatments and the minimum in the control treatment, as Glomeraceae was more abundant in the C. Our justification is contrary to Yao et al. (2022), who proposed that stress from heavy metals can improve the bacterial community’s capacity for functional adaptation.

This study demonstrated that heavy metal stress improved the endophyte community’s structural and functional plasticity and that keystone taxa significantly increased the effectiveness of phytoremediation. We can support our suggestion with the study of Mishra et al. (2019), who illustrated that because AMF can detoxify HMs-induced stress, it is regarded as one of the greatest biological strategies for boosting plant growth and shoot biomass. By immobilizing HMs in fungal structures, precipitating and chelating in the rhizosphere, sequestering HMs in vacuoles, and stimulating antioxidant mechanisms in the plants, AMF lessens HMs stress.

### 4.4 Correlations between micro plastic, HMs, shifts in soil microbial communities and soil physiochemical attributes

The association between environmental factors and microbial communities was assessed using redundancy analysis (RDA) and a matrix test with pairwise comparisons of soil indicators, with a colour gradient denoting Pearson’s correlation coefficient. Our studies showed that the environmental factors alter the microbial community. Soil physiochemical parameters also altered each other by showing a negative or positive correlation with each other (**Figures 6A, B, C**). The first two RDA dimensions showed a 68.41% variation in bacterial communities. Environmental factors such as ammonia, TN, Cu, EC, and As were positively correlated with RDA1. Furthermore, more abundance was also present at RDA1 (**Figure 6A**), and the first two RDA dimensions explained 35.91% of the variation in fungal communities. Environmental factors Zn, Cd, TC, pH, EC, TP, and OM were positively correlated with RDA1, and more abundance was also present in RDA1 (**Figure 6B**).

Mataruga et al. (2020) also found that the pH of the soil has a major impact on how well it binds to copper. The available soil Cu content dropped when the pH of the soil was increased and was in between 7-8. These findings support the current research that shows a negative correlation between soil pH and Cu content in the copper mine soil with the addition of PS-MPs. In our study, we also found synergistic and competitive interactions between soil physiochemical properties through Pearson correlation analysis (**Figure 6C**). According to earlier research, multiple contaminants can be absorbed by MPs (Ahmad et al., 2020; Zhao et al., 2022).

Obayomi et al. (2020) sated that pollutants in soil are tightly correlated with soil bacterial abundance, and MPs with varying particle sizes absorb different types and quantities of pollutants. Thus, we think that one element contributing to the diversification of microbial communities in MP-contaminated settings is the pollutants adsorbed by MPs. This study demonstrated that the varied distribution of microbial populations could be an ecological indicator for tracking the environmental health of soil.

## 5 Conclusion

This study determined that polystyrene microplastics (PS-MPs) and HMs affect soil physiochemical properties and *Lactuca sativa*. According to our results, the smaller size of PS-MPs was more lethal for the physio-biochemical attributes of the lettuce and soil. The toxicity of contaminants directly impeded the growth and altered the antioxidant activity of the lettuce. Our studies showed that the environmental factors alter the microbial community. In our study, we also found synergistic and competitive interactions between soil physiochemical properties through Pearson correlation analysis. This study demonstrated that the varied distribution of microbial populations could be an ecological indicator for tracking the environmental health of soil.

Our study showed that treatment with small MPs at the final point significantly increased bacterial and fungal diversity. Overall, bacterial diversity was higher in the rhizosphere, and fungal diversity was higher in the bulk soil. Therefore, our study confirms that microplastics size, different time zones, and spheres play an important role in the diversity of microbes. We noticed that the decrease in MPs size played an important role in decreasing AMF and increasing bacterial and fungal pathogens in the smaller MPs, especially in the rhizosphere community. Functional prediction was found significantly different in control, larger MPs from smaller PS-MPs. Hence, it was proved that HMs did not affect metabolic functions alone, possibly due to the abundant presence of AMF species in C and larger MPs. AMF may have played a role in the metabolic functions and has shown some resistance to the HMs by exhibiting a degree of tolerance to certain heavy metals. These fungi may have mechanisms to tolerate or accumulate heavy metals in their hyphae without experiencing detrimental effects. This study’s results will help to improve our understanding of the effects of PS-MPs and HMs on the soil and lettuce and provide important information for further studying the ecological risk of PS-MPs and HMs.

## Data Availability

The original contributions presented in the study are publicly available. This data can be found at the National Center for Biotechnology Information (NCBI) using accession number PRJNA1146469.
