# Supplementary material for: Micro plastic driving changes in the soil microbes and lettuce growth under the influence of heavy metals contaminated soil
Source: Front Plant Sci. 2024 Sep 11;15:1427166. doi: 10.3389/fpls.2024.1427166 (PMC11422782; doi:10.3389/fpls.2024.1427166)
Supplement: Supplementary file 1 [file DataSheet1.pdf]

Supplementary material

		Soil physiochemical Indicators							
**Treatments**	**pH**	**EC (µS/cm)**	**OM (%)**	**TP (mg/kg)**	**TC (mg/kg)**	**TN (mg/kg)**	**$NO3^-$ (mg/kg)**	**$NO2^-$ (mg/kg)**	**NH3 (mg/kg)**
**Day 0**	7.3733 ± .00577 a	763.6667 ± .57735 e	1.0100 ± 01732 a	104.4200 ± .36865 a	.5633 ± .01528 a	449.3333 ± .57735 d	.6133 ± .00577 b	.8233 ± .00577 b	2.5533 ± .00577 d
**C**	7.6133 ± .00577 d	824.6667 ± .57735 d	.8433 ± .03786 d	87.0067 ± .54629 e	.5033 ± .01155 c	501.6667 ± 57735 a	5767 ± .00577 c	.816 ± .00577 b	4.6567 ± .00577 a
**T1**	7.7167 ± .00577 c	952.0000 ± .00000 a	.9233 ±.02082 c	91.5700 ± .51449 d	.5367 ± .00577 b	476.6667 ± .57735 b	.9767 ± .00577 a	1.2867 ± .00577 a	4.3533 ± .00577 b
**T2**	7.7633 ± .00577 b	830.6667 ±. 57735 c	.9600 ± .0100 0 bc	99.8033 ± .60929 c	.5600 ± .01000 a	455.0000 ± .00000 c	.3600 ± .00000 d	.5000 ± .00000 c	3.0733 ± .00577 c
**T3**	7.7900 ± .00000 a	920.3333 ± .57735 b	.9700 ± .01000 b	102.9067 ± .29143 b	.5567 ± .00577 a	275.6667 ± .57735 e	.2767 ± .00577 e	.3433 ± .00577 d	2.2633 ± .00577 e

Table S1: Soil physiochemical properties at Mean ± SD and significant difference at ANOVA and Duncan's test. ($p < 0.05$) while C= control, T1= PS 106 µm, T2= 50 µm, T3= 13 µm.

	Treatments			
**Parameters**	**C**	**T1**	**T2**	**T3**
**Plant height (cm)**	29.1111 ± 8.20723 a	33.2556 ± 6.40861 a	15.9000 ± 3.80164 b	16.0444 ± 2.71897 b
**Whole plant weight (g)**	3.1789 ± 1.46683 b	5.2333 ± 1.76765 a	2.8278 ± .83664 bc	1.6000 ± .81360 c
**Leaf diameter (cm)**	4.2556 ± .33208 a	3.5111 ± .88380 b	3.1667 ± .48734 b	2.2333 ± .53619 c
**Leaf length (cm)**	11.7444 ± 1.01256 a	8.6667 ± 2.27651 b	8.0000 ± 1.30480 b	5.4889 ± 1.05053 c
**Leaves fresh weight (g)**	6.4267 ± 2.57450 a	3.8867 ± .30039 b	2.3433 ± .27791 b	1.4633 ± .41501 b
**Leaves dry weight (g)**	.2817 ± .07842 a	.3010 ± .07076 a	.2120 ± .06744 bc	.1447 ± .01069 c
**Electrolytic Leakage**	32.2200 ± 1.92258 a	33.6100 ± 3.75783 a	34.9170 ± 7.27058 a	37.3003 ± 11.25270 a
**Relative Water Content**	1.0460 ± .00100	.7497 ± .00006 b	.7053 ± .00058 c	.6373 ± .00058 d
**Stem Length (cm)**	13.8889 ± 5.05975 b	21.6778 ± 8.19768 a	12.6222 ± 4.28829 b	9.4556 ± 2.72218 b
**Stem fresh weight (g)**	1.1067 ± .54305 a	1.4144 ± .54925 a	.6389 ± .34002 b	.4478 ± .24662 b
**Stem dry weight (g)**	.2273 ± .07366 ab	.2920 ± .12020 a	.1010 ± .04681 bc	.0610 ± .02307 c
**Root Length (cm)**	7.5333 ± 1.41951 ab	8.6333 ± 1.46031 a	6.6667 ± 1.00995 b	5.1444 ± 1.46979 c
**Root fresh weight (g)**	.1922 ± .08511 b	.3367 ± .09552 a	.1800 ± .04555 b	.1900 ± .21720 b
**Root dry weight (g)**	.0757 ± .01779 b	.1553 ± .02250 a	.0617 ± .00907 bc	.0390 ± .00794 c
**SOD (U mg-1 protein)**	1.7382 ± .13387 a	1.2257 ± .05046 b	1.0241 ± .09352 c	.9197 ± .05694 c
**POD (ΔOD470/min/g)**	74.1333 ± 2.60256 d	116.1333 ± 20.33847 c	162.9333 ± 18.60036 b	339.4667 ± 18.13872 a

Table S2: Plant parameters at Mean ± SD at ANOVA and Duncan's test. ($p < 0.05$); while C= control, T1= PS 106 µm, T2= 50 µm, T3= 13 µm

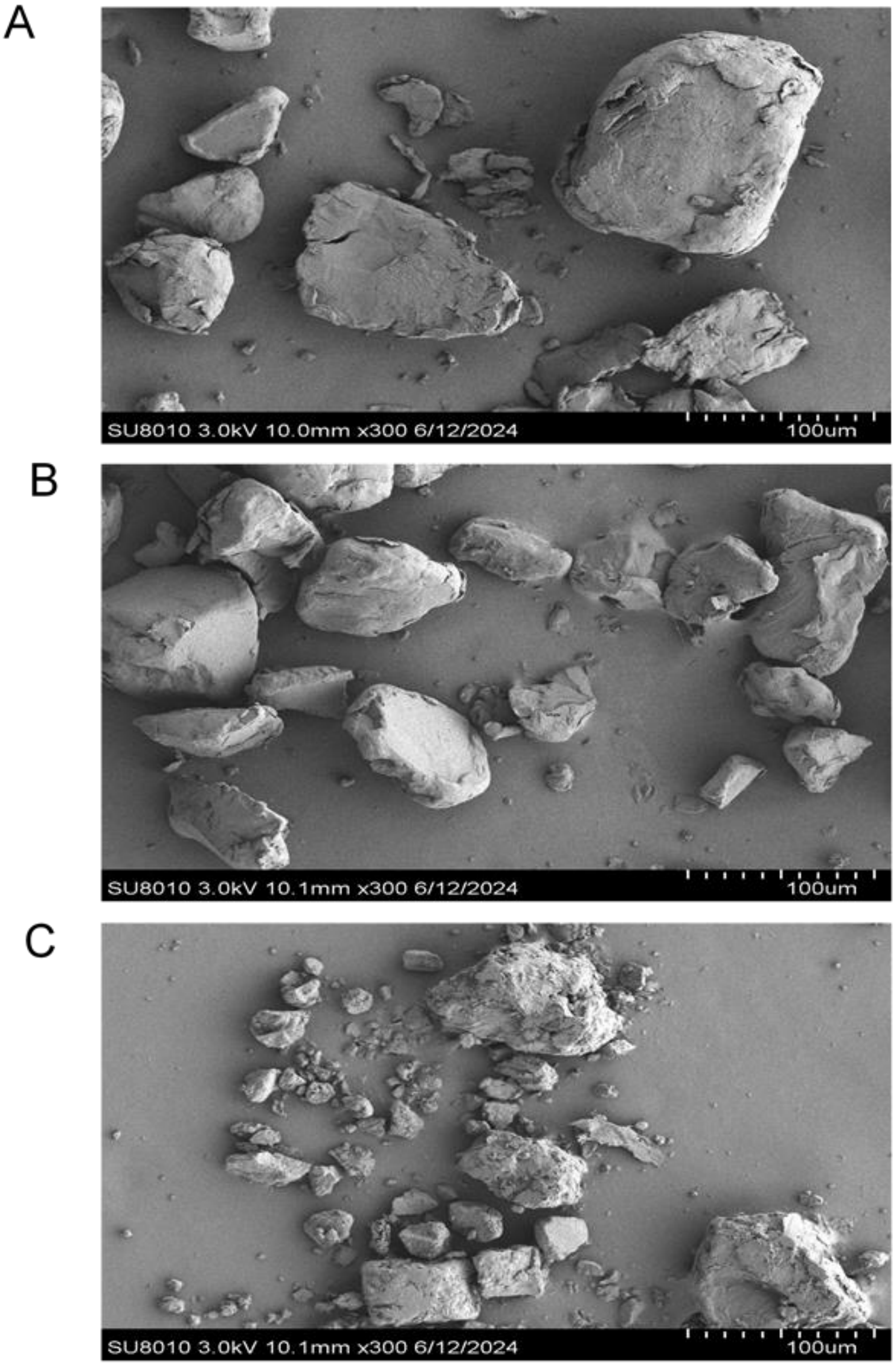

Figure S1. SEM images of PS-MPs indicate that PS-MPs are in nonuniform size and shape, and PS-MPs have blunt edges and rough surface. 10 mg of PS-MPs were taken by a spatula and poured onto a double-sided carbon tape, blown by a nitrogen gun to remove unstuck particles, then coated with sputtered ions for 40s at 25 mA (Emitech K575 Au-Pd sputter coater, Emitech Ltd., UK) for SEM observation, using a Hitachi SU8010 (Hitachi High-Tech America Inc., USA). Images were obtained in high vacuum mode with secondary electron detection at an accelerating voltage of 3 kV. While Figure A = 106 µm (T1), Figure B = 50 µm (T2) and Figure C= 13 µm (T3).

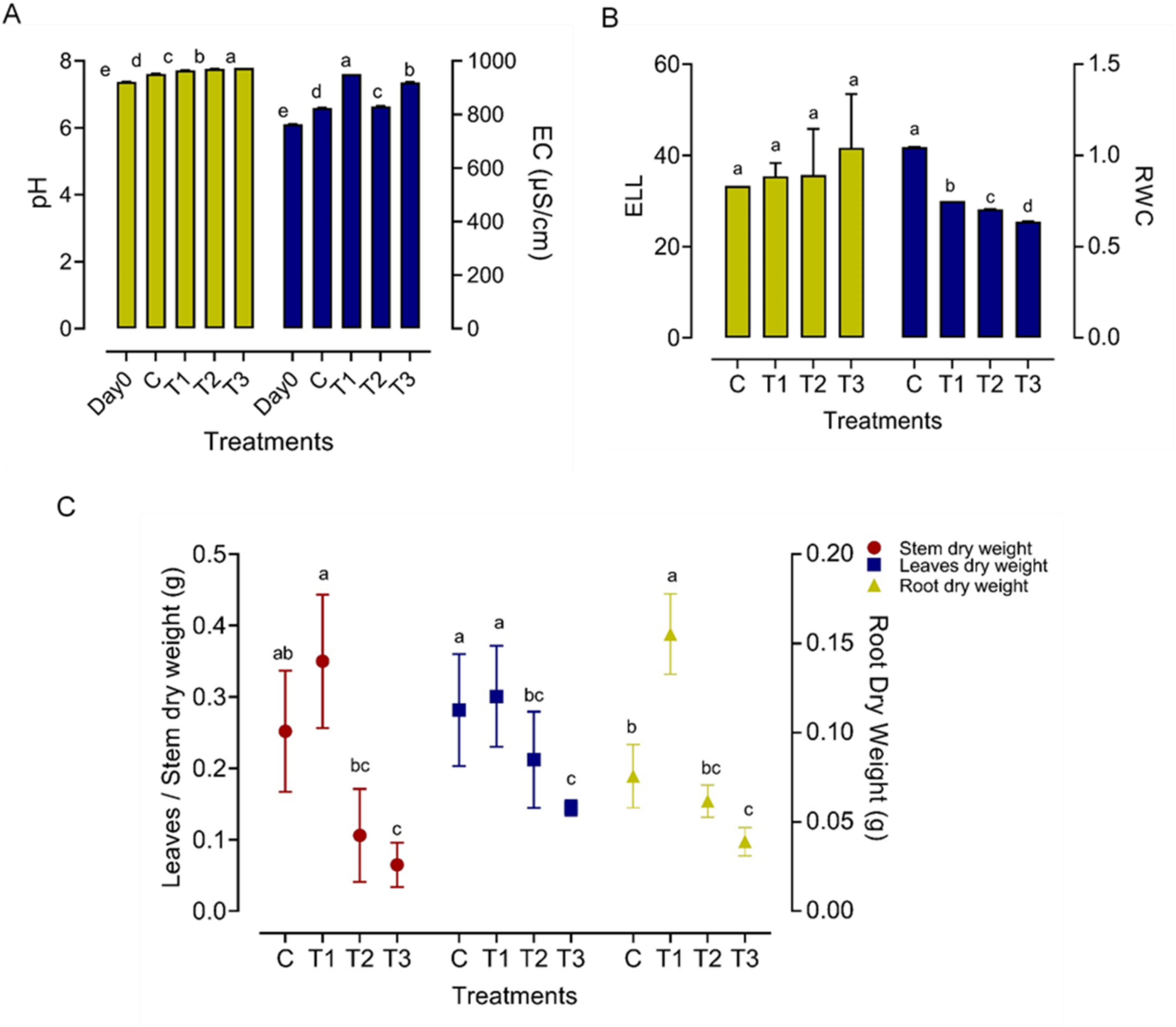

Figure S2: Plant physical parameters and soil chemical parameters. Figure A represents soil electric conductivity (EC), and soil pH. Figure B represents the relative water content of leaves (RWC) and electrolytic leakage of leaves (ELL) and Figure C represents the dry weight of leaves, dry weight of root, and dry weight of the stem. Different letters indicate significant differences at $P \leq 0.05$ while C control, T1= PS 106 µm, T2= 50 µm, and T3= 13 µm.

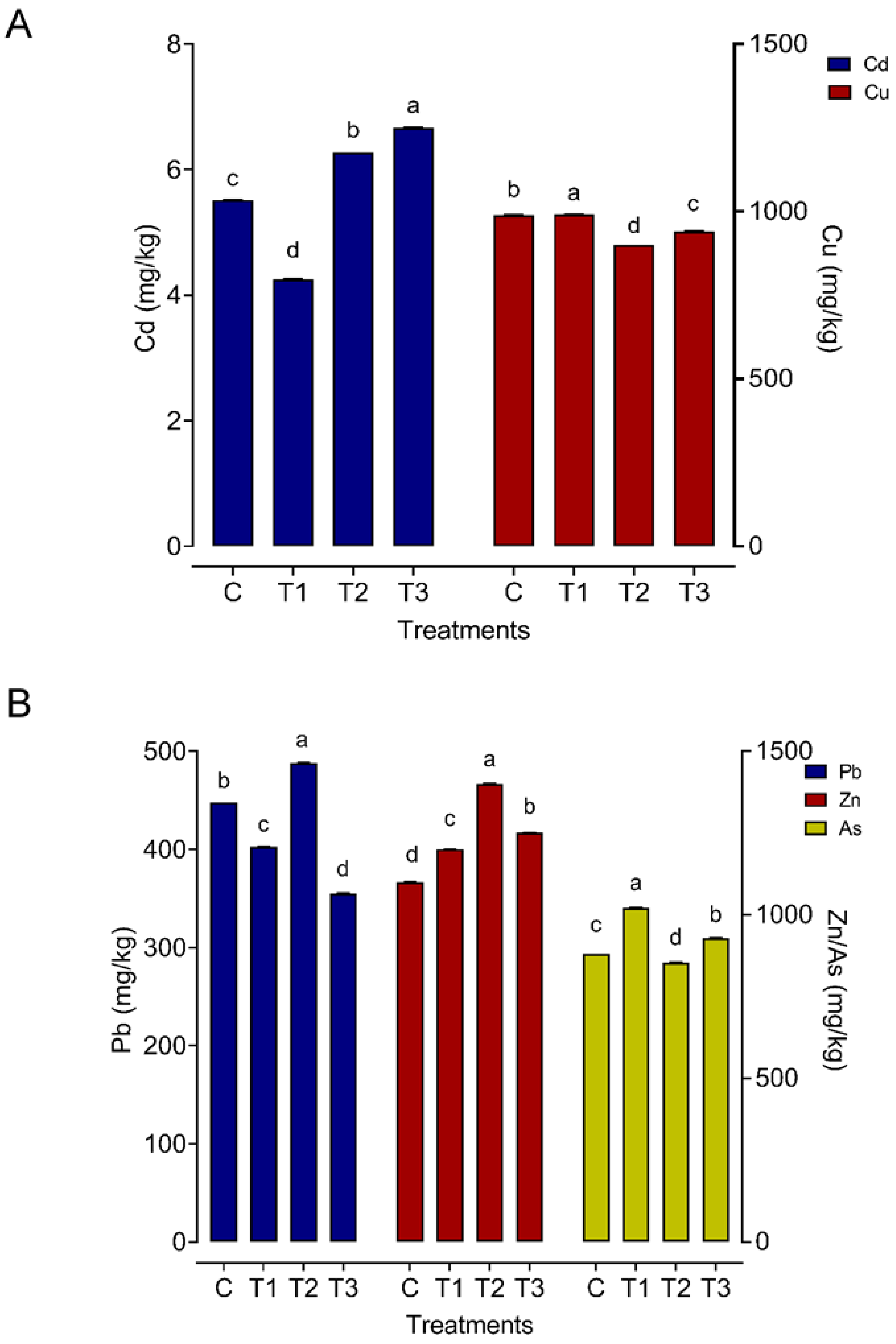

Figure S3: Soil heavy metals. Figure A represents Cd (cadmium) and Cu (cupper). Figure B represents Pb (iron), Zn (zinc) and As (arsenic). Different letters indicate significant differences at $P \leq 0.05$ while C= control, T1= PS 106 µm, T2= 50 µm and T3= 13 µm.

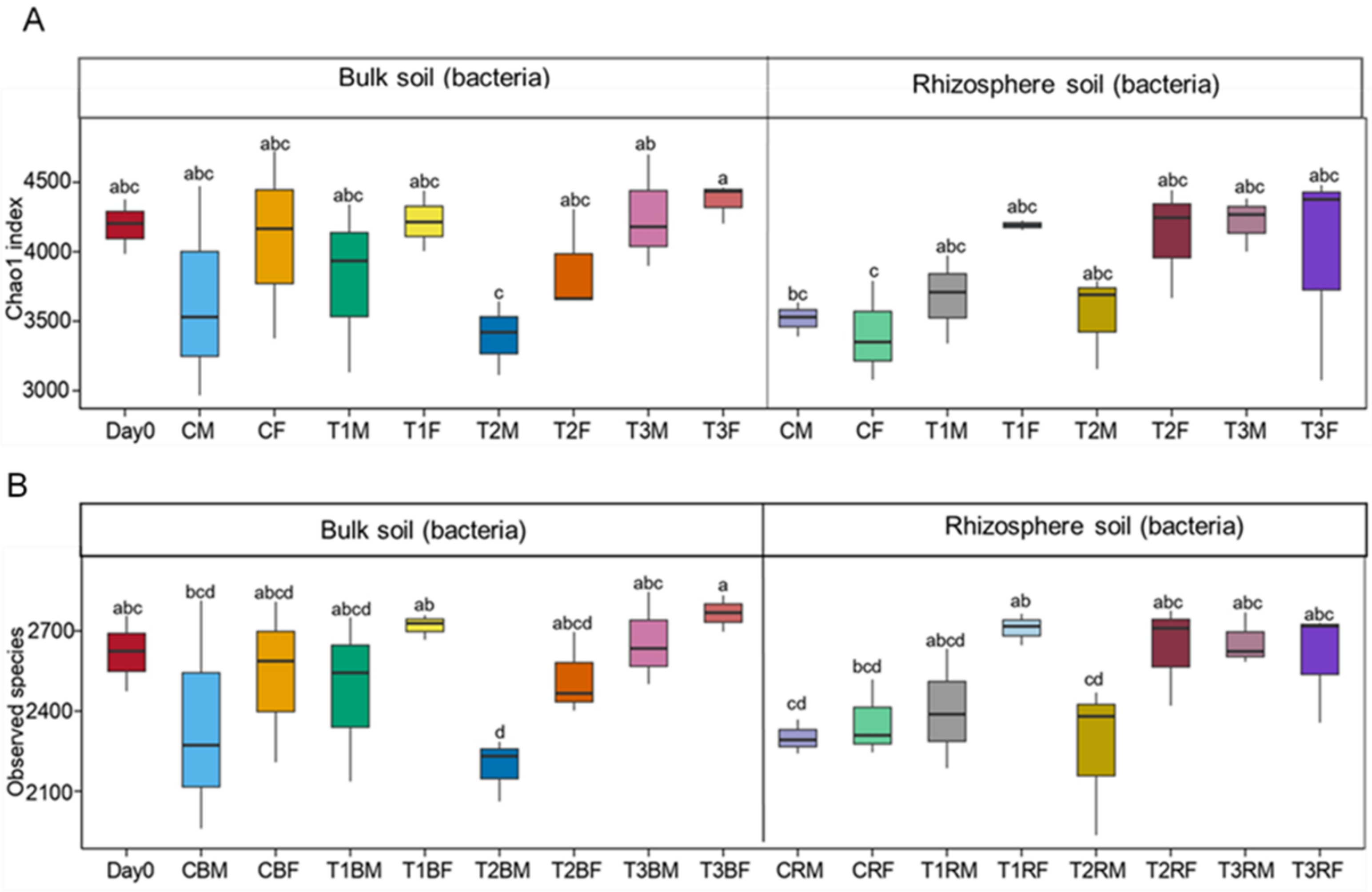

Figure S4: Alpha diversity of soil bacterial community. Figure A represents Chao1 index of bulk and rhizosphere soil of bacterial community. Figure B represents Observed species of bulk and rhizosphere soil of fungal community; while C= control, T1= PS 106 µm, T2= 50 µm, T3= 13 µm, B= bulk soil, R= rhizosphere soil, M= middle point and F= final point.

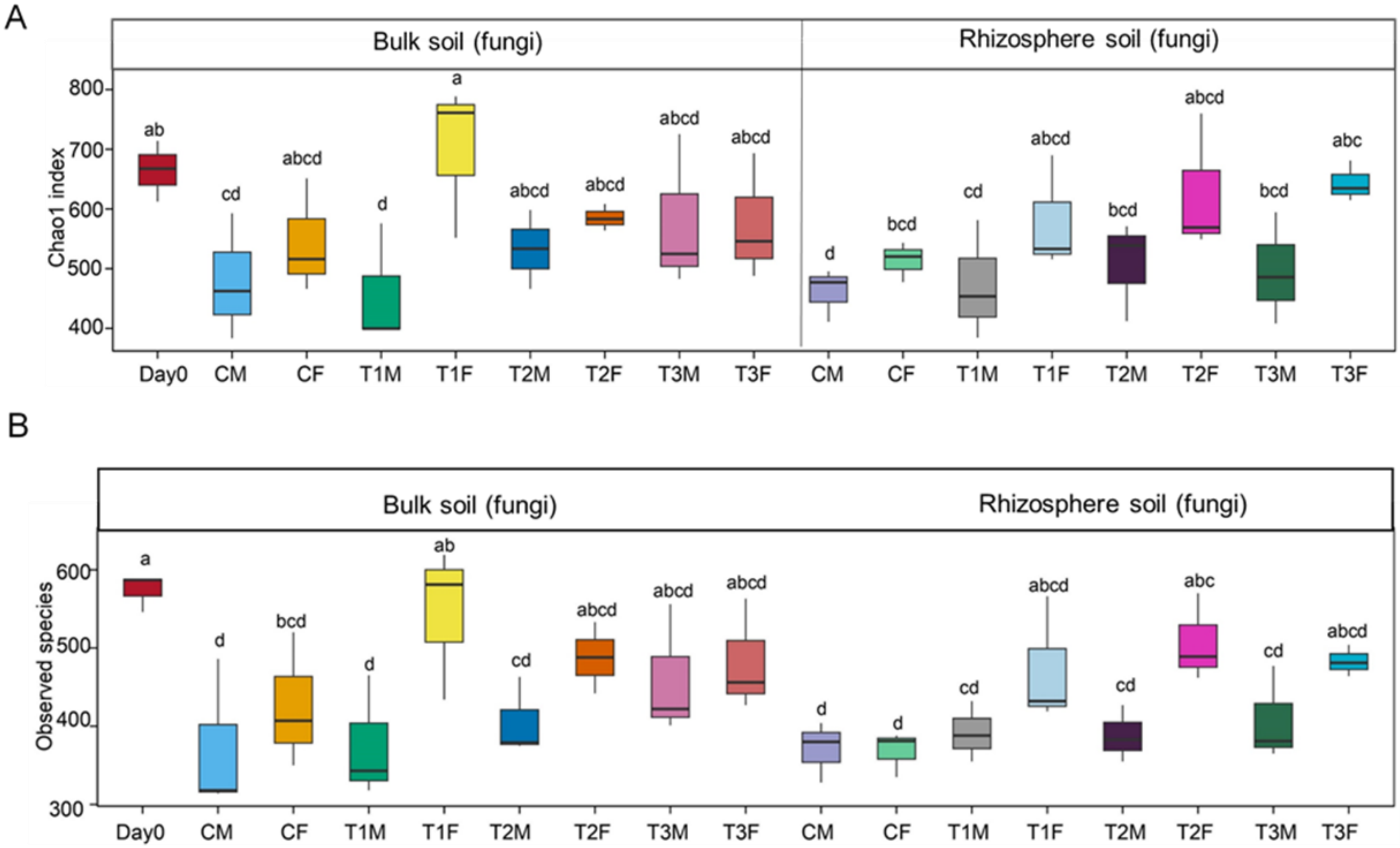

Figure S5: Alpha diversity of soil fungal community. Figure A represents Chao1 index of bulk and rhizosphere soil of fungal community. Figure B represents observed species of bulk and rhizosphere soil of fungal community; while C= control, T1= PS 106 μm, T2= 50 μm, T3= 13 μm, B= bulk soil, R= rhizosphere soil, M= middle point and F= final point.
